# Electron-rich triarylphosphines as nucleophilic catalysts for oxa-Michael reactions

**DOI:** 10.3762/bjoc.17.117

**Published:** 2021-07-21

**Authors:** Susanne M Fischer, Simon Renner, A Daniel Boese, Christian Slugovc

**Affiliations:** 1 Institute for Chemistry and Technology of Materials, Graz University of Technology, Stremayrgasse 9, 8010 Graz, Austria; 2 Christian Doppler Laboratory for Organocatalysis in Polymerization, Stremayrgasse 9, 8010 Graz, Austria,; 3 Physical and Theoretical Chemistry, Institute of Chemistry, University of Graz, Heinrichstrasse 28/IV, 8010 Graz, Austria

**Keywords:** Michael acceptor affinity, Michael addition chemistry, organocatalysis, phosphine, solvent-free synthesis

## Abstract

Electron-rich triarylphosphines, namely 4-(methoxyphenyl)diphenylphosphine (MMTPP) and tris(4-trimethoxyphenyl)phosphine (TMTPP), outperform commonly used triphenylphosphine (TPP) in catalyzing oxa-Michael additions. A matrix consisting of three differently strong Michael acceptors and four alcohols of varying acidity was used to assess the activity of the three catalysts. All test reactions were performed with 1 mol % catalyst loading, under solvent-free conditions and at room temperature. The results reveal a decisive superiority of TMTPP for converting poor and intermediate Michael acceptors such as acrylamide and acrylonitrile and for converting less acidic alcohols like isopropanol. With stronger Michael acceptors and more acidic alcohols, the impact of the more electron-rich catalysts is less pronounced. The experimental activity trend was rationalized by calculating the Michael acceptor affinities of all phosphine–Michael acceptor combinations. Besides this parameter, the acidity of the alcohol has a strong impact on the reaction speed. The oxidation stability of the phosphines was also evaluated and the most electron-rich TMTPP was found to be only slightly more sensitive to oxidation than TPP. Finally, the catalysts were employed in the oxa-Michael polymerization of 2-hydroxyethyl acrylate. With TMTPP polymers characterized by number average molar masses of about 1200 g/mol at room temperature are accessible. Polymerizations carried out at 80 °C resulted in macromolecules containing a considerable share of Rauhut–Currier-type repeat units and consequently lower molar masses were obtained.

## Introduction

**Scheme 1:**
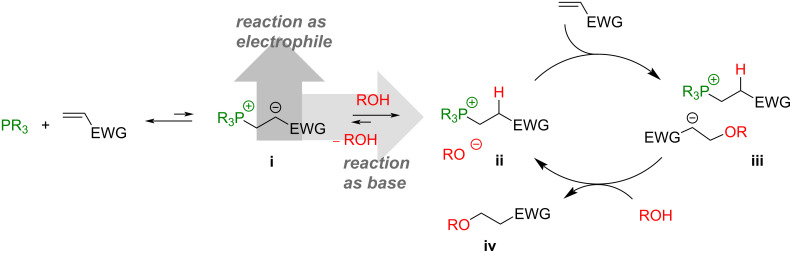
Mechanism for the phosphine-initiated oxa-Michael addition.

Phosphines are potent nucleophiles that are used as catalysts in many reactions, like Rauhut–Currier, Morita–Baylis–Hillman or Michael reactions [1–3]. The first step of these reactions is a conjugate addition of the phosphine to an activated electrophile, e.g., an electron-deficient olefin, generating a zwitterion (**i**, Scheme 1). In further course, the zwitterion acts as a nucleophile or as a base [1]. The efficiency of the formation of this β-phosphonium α-carbanionic species depends on the nucleophilicity of the phosphine which is usually stronger in trialkylphosphines and decreases with aryl substitution [4–5]. Consequently, the first phosphine-catalyzed reactions have been described with trialkylphosphines [6–10]. However, trialkylphosphines are characterized by a pronounced oxidation sensitivity demanding the exclusion of oxygen. This issue can be mitigated by using triarylphosphines that are by far less prone to oxidation. Both, the rate of oxidation and the reactivity in nucleophilic additions correlate with the electron density residing on the phosphorous center [11–13]. Accordingly, triarylphosphines are generally less reactive in conjugate additions than trialkylphosphines and often high catalyst loadings of up to 20 mol % and elevated temperatures are necessary to obtain satisfactory conversions [5,14–15]. The low reactivity of arylphosphines can be enhanced by introducing electron-donating groups (e.g., -CH_3_, -OMe, -NMe_2_) at the aryl moieties. In this way, the electron density on the phosphorous and thus the nucleophilicity is increased. This strategy has for example been exploited in the reaction of ethyl acrylate with 4-nitrobenzaldehyde [16], in aza-Morita–Baylis–Hillman reactions [17], or in umpolung [3 + 2] annulations [18]. In all these cases, the reactions were performed without protective gas indicating that electronically modified arylphosphines tolerate the presence of oxygen.

Herein we wish to report the scope of three different triarylphosphine catalysts in the oxa-Michael addition. Triphenylphosphine (TPP), (4-methoxyphenyl)diphenylphosphine (MMTPP) and tris(4-trimethoxyphenyl)phosphine (TMTPP). The catalysts were investigated in the reaction of four different Michael acceptors with four different alcohols. In the oxa-Michael addition, the zwitterion **i**, initially formed by the conjugate addition of the phosphine to the Michael acceptor, is believed to be protonated by the alcohol forming the actual catalytically active species namely ion pair **ii**, consisting of a phosphonium cation and an alkoxide. The alkoxide in **ii** then reacts with another electrophile generating the ion pair **iii**. In the final step, the α-carbanionic species in **iii** gets protonated by an alcohol generating the oxa-Michael addition product (**iv**) and regenerating **ii** (Scheme 1). Additionally, the ion par **ii** might directly react via a nucleophilic substitution of the phosphonium group by the alkoxide to yield the product **iv** and the phosphine. Our results disclosed in the following contribute to the rational selection of proper (pre-)catalysts for this and similar reactions also considering the oxygen sensitivity of the nucleophiles.

## Results and Discussion

**Figure 1:**
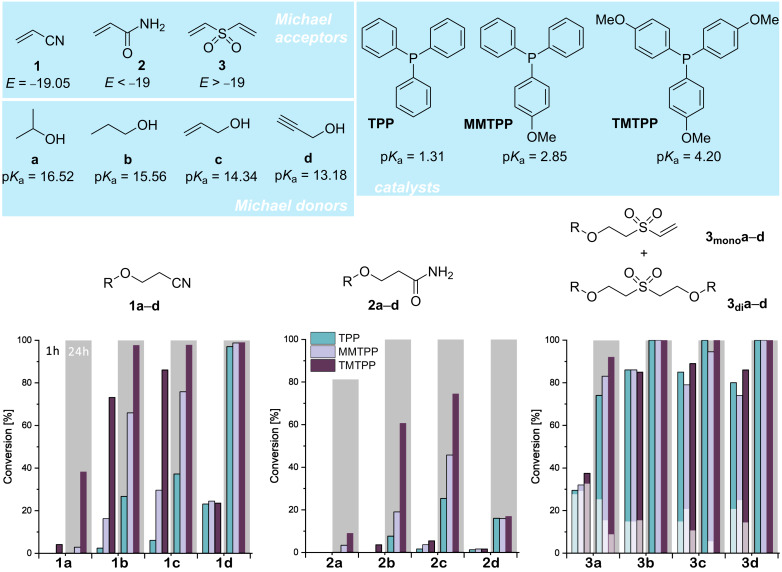
Above: Michael acceptors, Michael donors and catalysts used in this study; p*K*_a_ (respectively p*K*_a_ of the conjugated acid in case of phosphines) calculated using the p*K*_a_ prediction platform (neural network result for solvent H_2_O) available at pka.luo-group.com [20]; below: Conversion of the oxa-Michael reaction of acrylonitrile (left), acrylamide (middle), and divinyl sulfone (right; double bond conversion is given; light sections of the bar represent the share of **3_mono_a**–**d**, dark sections represent the share of **3_di_a**–**d**) with the alcohols propan-2-ol (**a**), propan-1-ol (**b**), prop-2-en-1-ol (**c**), and prop-2-yn-1-ol (**d**) catalyzed by triphenylphosphine (TPP), (4-methoxyphenyl)diphenylphosphine (MMTPP), and tris(4-methoxyphenyl)phosphine (TMTPP). Reaction conditions: 1 equiv Michael acceptor, 2 equiv alcohol (in case of **3**, 3 equiv alcohol), 1 mol % catalyst (with respect to the Michael acceptor), 1 h and 24 h (bars above grey boxes) at room temperature (23 °C); no solvent used.

To compare the activity of the triarylphosphines TPP, MMTPP and TMTPP as catalysts for the oxa-Michael reaction three varyingly strong Michael acceptors, namely acrylonitrile (**1**), acrylamide (**2**) and divinyl sulfone (**3**) were reacted with four different alcohols of similar molecular mass but different acidity (Figure 1). The stoichiometry of Michael acceptor to alcohol was set to 1 to 2 and no additional solvent was used. The reaction was carried out at room temperature with 1 mol % catalyst (with respect to the Michael acceptor). The reaction progress was monitored after 1 h and 24 h using ^1^H NMR spectroscopy. The set-up of the study aims to show the scope and the limitations of the different catalysts. An optimization of the reaction conditions in terms of obtaining full conversion in the shortest time possible with the lowest reasonable achievable catalyst loading was not undertaken. The results are shown in Figure 1. The benchmark catalyst TPP is unable to promote the oxa-Michael reaction of the good Michael acceptor **1** (electrophilicity parameter *E* of −19.05 [19]) with the least acidic alcohol 2-propanol (**a**) as virtually no conversion was observed after 24 h. Using MMTPP leads to a minor improvement and a 3% conversion towards **1a** was found after 24 h. TMTPP, however, gives already 4% conversion after 1 h and 38% conversion after 24 h. The more acidic 1-propanol (**b**) readily reacts in the presence of TPP (27% conversion after 24 h). MMTPP already provides a considerable improvement since a conversion of 66% is obtained after 24 h but TMTPP is again a distinctly better catalyst providing 73% conversion after 1 h and almost full conversion (98%) after 24 h. Allyl alcohol (**c**) is more reactive than 1-propanol as conversions with all catalysts at all conditions are slightly higher. Most importantly, the TMTPP-catalyzed reaction shows already 86% conversion after 1 h. In sharp contrast, propargyl alcohol (**d**), the most acidic one, gave only about 24% conversion after 1 h irrespective of which catalyst had been used. After 24 h almost full conversion (97% TPP or 99% MMTPP and TMTPP) was found for all three catalysts. Accordingly, in this case, the activity of the catalyst is not rate determining. This observation is rationalized by the occurrence of a non-productive acid–base equilibrium involving the de- and re-protonation of the considerably acidic alkyne proton in **d** (p*K*_a_ = 15.61 [20]) [21]. The reaction conditions disclosed here are an improvement compared to the state of the art. For example, addition product **1c** has been obtained in 93% conversion before using 10 mol % TPP, 3 equiv **c** and heating the reaction mixture for 8 h under refluxing conditions [14]. However, with base catalysis (KO*t*-Bu) even better results than those presented here can be achieved [22–23].

Switching to the weaker Michael acceptor acrylamide (*E* = −23.54 for *N*,*N*-dimethylacrylamide) [19], no useful conversions on any account were obtained. However, TMTPP performs best, giving 61 and 74% conversions with 1-propanol (**b**) and allyl alcohol (**c**) after 24 h. To illustrate that the reaction does not stop after 24 h the conversions were re-checked after 21 d. After this time with TMTPP as the catalyst, conversions of 44% (**3a**), 92% (**3b**), 98% (**3c**), and 91% (**3d**) are obtained. No indications for aza-Michael reactions potentially leading to polyamide 3 like structures were observed [24]. A more efficient transformation of acrylamide can be obtained with base catalysis. Using activated potassium carbonate, a reaction temperature of 40 °C, and 4 h reaction time give typically better conversions than those reported herein with nucleophiles [25].

Next, the difunctional divinyl sulfone was tested as the strongest Michael acceptor (*E* = −18.36, for phenyl vinyl sulfone [19]) under investigation. In distinction from the experiments described above, three equivalents of the alcohol were used. In general, the different catalysts perform very similar in this reaction giving high double-bond conversions of about 80% after already 1 h [26]. A mixture of mono- (**3_mono_a**–**d**) and di-adducts (**3_di_a**–**d**) are observed and only in case of 2-propanol also divinyl sulfone is still present. With 2-propanol a slight but significant influence of the catalyst choice on the conversion is observed (Figure 1). With all other (more acidic) alcohols, the conversion is reaching completeness after 24 h. Why MMTPP is performing slightly worse than TPP as indicated by the double-bond conversion and by the higher share of the mono-adduct **3_mono_a**–**d** after 1 h reaction time is not clear. The reaction of **3** with 3 equiv **a** or **c** catalyzed with 10 mol % TPP at 40 °C using dichloromethane ([DVS] = 0.55 M) as solvent has been described. The product **3a** was obtained as a 76:13 mixture of **3_mono_a** and **3_di_a** and **3c** as a 11:89 mixture of **3_mono_c** and **3_di_c** [21]. The herein disclosed results highlight that solvent-free conditions are particularly effective and allow for reducing the catalyst loading by the factor of 10, thereby obtaining a higher share of **3_di_a** and full conversion towards **3_di_c**. Interestingly, the catalytic activity of TPP in reactions with **3** as the Michael acceptor is only slightly lower than the activity of the methoxy-substituted congeners.

**Figure 2:**
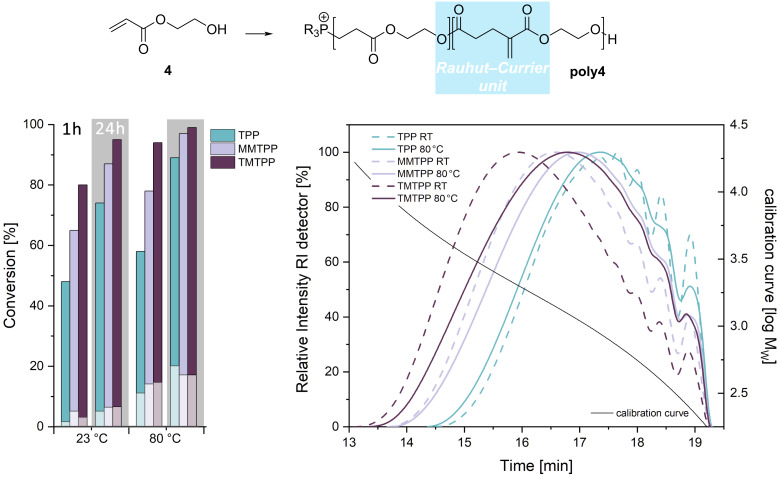
Left: double-bond conversion of the polymerization of **4** initiated by 5 mol % TPP, MMTPP or TMTPP after 1 h at room temperature (23 °C) and at 80 °C as well as after 24 h at 23 °C and at 80 °C; light sections of the bars represent the share of Rauhut–Currier repeat units; right: size exclusion chromatograms (in THF, relative to poly(styrene) standards) of **poly4** prepared with 5 mol % TPP, MMTPP or TMTPP using a reaction time of 24 h and a reaction temperature of 23 °C (dashed lines) or 80 °C (full lines).

As an example for acrylates as Michael acceptors, the performance of the catalysts in the oxa-Michael addition polymerization of 2-hydroxyethyl acrylate (HEA, **4**) was investigated [27–29]. The catalyst loading was increased to 5 mol %, because 1 mol % was not sufficient to obtain satisfying conversions. The reaction mixture consisting of **4** and the catalyst was either stirred at room temperature or put in a drying chamber operated at 80 °C. Aliquots of the reaction mixture were sampled after 1 and 24 h and analyzed by ^1^H NMR spectroscopy and size exclusion chromatography (SEC). The results are shown in Figure 2.

After 1 h at room temperature, an impact of the catalysts on the double bond conversion is evident. TPP gave a double bond conversion of 48%, while MMTPP and TMTPP performed better with 67 and 80%, respectively. After 24 h at room temperature conversions increased to 74% (TPP), 85% (MMTPP), and 90% (TMTPP). Performing the reaction at 80 °C leads to higher double-bond conversions than reactions run at room temperature. After 1 h reaction time conversions of 58% (TPP), 78% (MMTPP), and 94% (TMTPP) were obtained. Prolonging the reaction time to 24 h led to high double-bond conversion of 89% in case of TPP and 97% and 99% in the cases of MMTPP and TMTPP. Molar mass distributions of the polymers prepared with a reaction time of 24 h were determined by SEC. First, the polymerizations conducted at room temperature are discussed. As expected from the trend in double-bond conversion, the number average molar mass (*M*_n_) of **poly4** increases according to the activity of the initiator. The *M*_n_ values nearly doubled when going from TPP (660 g/mol, dispersity *Ð* = 1.5) to TMTPP (1160 g/mol, *Ð* = 1.8) with MMTPP (910 g/mol, *Ð* = 1.7) lying in about the middle of these two values. Turning to the results obtained for the polymerization conducted at 80 °C it is revealed that **poly4** prepared with TPP is characterized by only a slightly higher *M*_n_ value of 680 g/mol than **poly4** from the room temperature reaction. MMTPP and TMTPP derived **poly4** exhibiting even lower *M*_n_ values (820 and 890 g/mol, *Ð* = 1.7 and 1.8) than those obtained in the room temperature reaction. Considering the distinctly higher double-bond conversions at 80 °C, these findings point to another double-bond consuming reaction beside the oxa-Michael reaction. The evaluation of the NMR spectra indicate, among repeating units from oxa-Michael and transesterification reactions [30–31], the presence of Rauhut–Currier-derived linkages [32–34]. This repeat unit is characterized by peaks at 6.22 and 5.64 ppm in the ^1^H NMR spectrum and at 126.6, 33.0, 27.3 ppm in the ^13^C NMR spectrum of **poly4** (see Supporting Information File 1) and its share is with approximately 17–20% higher in polymers prepared at 80 °C (Figure 2). The formation of this repeat unit consumes two equivalents of acrylates and thus, disproportionally decreases the quantity of acrylate groups in relation to alcohol groups. Consequently, the originally ideal stoichiometry of Michael acceptors and Michael donors is changed in favor of alcohols. This eventually results in lower molecular mass distributions in cases in which more Rauhut–Currier repeat units are formed. In comparison, **poly4** has been prepared with nucleophilic catalysis using 10 mol % N-heterocyclic carbenes such as 1,3,4-triphenyl-4,5-dihydro-1*H*-1,2,4-triazol-5-ylidene or 1,3-bis(2,6-diisopropylphenyl)imidazol-2-ylidene. The polymerization was carried out at room temperature for 24 h and no solvent was used. The resulting reaction mixture was dissolved in dichloromethane and precipitated from diethyl ether resulting in about 50% polymer yield featuring *M*_n_ values of 1500–1800 g/mol [30].

**Figure 3:**
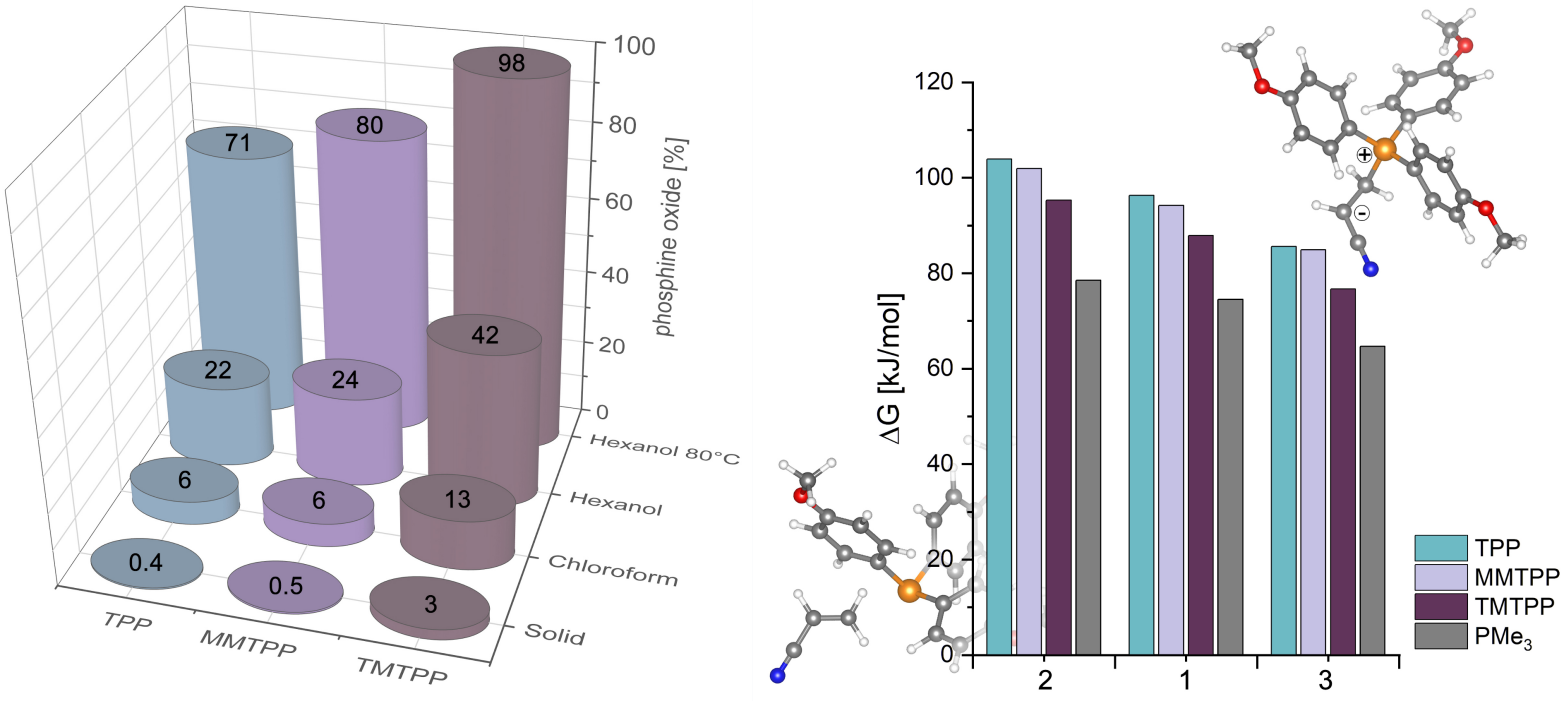
Left: Oxidation stability of the phosphines. Phosphine oxide content in % as determined by ^31^P NMR spectroscopy after a 14 d exposure to air under the following conditions: sample stored as a solid at room temperature, samples dissolved in chloroform and 1-hexanol (stored at room temperature in the dark), and in 1-hexanol (stored at 80 °C in the dark). Right: Relative stabilities of the zwitterions formed upon reaction of Michael acceptors **1**–**3** with the phosphines (the cartoon shows the structures of the educts acrylonitrile and TMTPP (left, behind the chart) and the corresponding zwitterion (right, above), optimized at the B3LYP/def2-TZVPPD level of theory).

Next, the oxidation stability of the catalysts was tested. For this purpose, the three different phosphines were exposed to air for 14 d in dark conditions. Four different conditions were chosen. Undissolved solid samples and samples dissolved in chloroform or in 1-hexanol were kept at room temperature and solutions in 1-hexanol were also heated at 80 °C. The reaction mixture was then investigated via ^31^P NMR spectroscopy. Under all conditions, the formation of the corresponding phosphine oxide derivative as the only decomposition product was observed. The results, shown in Figure 3, reveal that the oxidation stability is decreasing in the order TPP > MMTPP > TMTPP, which is in line with electrochemical studies showing a decrease of the oxidation potential from 1.400 V (TPP) to 1.050 V (TMTPP) [35].

Furthermore, the share of phosphine oxide is dependent on the oxygen solubility in the solvent, as indicated by the experiments in chloroform and 1-hexanol exhibiting the higher oxygen solubility [36]. To obtain further insight, the SOMO energies of the radical cations of the phosphines under investigation were calculated by density functional theory (DFT), namely B3LYP-def2-TZVPPD. According to criterion introduced by Stewart et al. postulating air stability of phosphines when the SOMO energy is higher than −10 eV, the three derivatives should be air stable [13]. However, the SOMO energies decrease within the series from −9.60 eV (TPP, −9.50 according to [13]) to −9.18 (MMTPP) and −8.59 (TMTPP) suggesting TMTPP to exhibit the highest oxidation stability within the series; the opposite what was observed experimentally. Therefore, the oxidation stability of the phosphines discussed here cannot be described by evaluating their SOMO energies as suggested previously. Overall, the experiments demonstrate that the oxidation stability of all phosphines under investigation can be considered sufficient for running reactions (under typically employed reaction conditions, i.e., reaction temperatures and times not exceeding 80 °C and 24 h) without the unconditional need to exclude oxygen.

A first hint for rationalizing the different reactivity of the different phosphines can be retrieved from the p*K*_a_ value of their conjugated acids. Substitution of the aromatic rings with methoxy groups increases the p*K*_a_ value from 1.31 (TPP) to 4.20 (TMTPP) (Figure 1). Methyl cation affinities (MCA) which can be used as descriptors for the nucleophilicity of a compound were calculated by Lindner et al. who suggested TMTPP (651.0 kJ/mol) to be a stronger Lewis base than TPP (618.7 kJ/mol) [37]. However, for PMe_3_, discussed as a model for aliphatic phosphines, a distinctly lower MCA of 604.2 kJ/mol was calculated. This is in contrast to experimental data as PMe_3_ is known as a more active catalyst for oxa-Michael additions than arylphosphines [8,14]. Apparently, the MCA is not correlating with the phosphines’ activities in conjugate addition reactions. Another approach for assessing the nucleophilicity of the phosphines is to compare their HOMO energy. The nucleophilicity should decrease with increasing s character of the orbital containing the lone pair, which should also be the HOMO of the molecule. A higher s character of the HOMO, going in hand with a lower energy level of the HOMO, is thus indicative for a lower nucleophilicity [38–39]. Accordingly, the HOMO energies have been calculated and increase from −5.91 eV (TPP) to −5.73 eV (MMTPP) and −5.42 eV (TMTPP). A comparison of the orbital distributions of the arylphosphines reveals that the HOMO of all phosphines under investigation has a significant phosphorous character (visual representations are provided in Supporting Information File 1). However, considering the HOMO energy of PMe_3_ which is calculated to be as low as −6.10 eV, it is obvious that also this approach fails in sufficiently describing the activity of phosphines in catalyzing oxa-Michael reactions. To resolve this issue, the Gibbs free energy (Δ*G*) of the reaction of TPP, MMTPP, TMTPP, and PMe_3_ with acrylonitrile leading to zwitterion formation (Figure 3, right) was calculated in chloroform. The Michael acceptor affinity (MAA) of the nucleophiles is then given by the Gibbs free energy of the back reaction [37]. The respective energy differences calculated at the B3LYP/def2-TZVPPD level of theory are −96.3 kJ/mol (TPP), −94.2 kJ/mol (MMTPP), −87.9 kJ/mol (TMTPP), and −74.5 kJ/mol (PMe_3_) in favor of the educts acrylonitrile and phosphine. Accordingly, the zwitterion formed from PMe_3_ is in relation the most stable and the zwitterion formed from TPP the most unstable one within the series. The stability trend of the zwitterions based on acrylamide and divinyl sulfone is the same (Figure 3, right). The different reactivity of the three Michael acceptors is apparent from the relative stabilities of the zwitterion. Acrylamide gives the least stable (MAA with TPP is −103.9 kJ/mol) and DVS the most stable zwitterion (MAA with TPP: −85.6 kJ/mol). Consequently, such calculated Michael acceptor affinities correlate with the experimental results and are suited to reflect the actual activity of the phosphines under investigation. This is reasonable because the position of the thermodynamic equilibrium of the unreacted Michael acceptor and -donor and the corresponding zwitterion **i** is believed to be decisive for the efficacy of the subsequent reaction, protonation of **i** by the alcohol resulting in the formation of ion pair **ii** (Scheme 1) [40]. In turn, the p*K*_a_ value of the alcohol is another important parameter for the speed of the overall reaction. The alcohol’s acidity is determining how efficiently **i** is transformed into the ion pair **ii** (Scheme 1) being the actual entry point into the catalytic cycle of the oxa-Michael reaction. Accordingly, the reactivity trend observed for the different alcohols under investigation is rationalized. Note that although a two-step process is discussed herein, it is also conceivable that the reaction towards **ii** proceeds via a single transition state involving the Michael acceptor, the Michael donor, and the alcohol. Furthermore, the different nucleophilicity of the generated alkoxides might play an additional role. However, it has been shown, that the nucleophilicity of alkoxides differs only moderately [41]. Therefore, this effect is considered to be less important for the explanation of the relative characteristics of the reactions than the factors discussed above.

## Conclusion

The activity of differently substituted triarylphosphines in the oxa-Michael addition of alcohols to electron-deficient olefins was investigated. In general, the activity increases with increasing methoxy-substitution in the order TPP < MMTPP < TMTPP. The activity order was rationalized based on DFT calculations by an increasing stationary concentration of the primary reaction product, the corresponding β-phosphonium α-carbanionic zwitterion, when using arylphosphines with more electron-donating substituents. Besides the catalyst, the second decisive factor for the speed of the reaction is the acidity of the alcohol as the efficacy of the secondary reaction, where the zwitterion reacts with the alcohol, increases when more acidic alcohols are used. Moreover, concentrated conditions or the omission of solvents is beneficial for this reaction. In summary, the better catalyst TMTPP is particularly useful for reacting weak Michael acceptors and/or less acidic alcohols. Phosphine loadings of only 1 mol % with respect to the Michael acceptor are in many cases sufficient to provide a full conversion within 24 h at room temperature. With good Michael acceptors and/or acidic alcohols the catalytic activity of TPP becomes competitive to the one of the more expensive TMTPP. Furthermore, TMTPP is somewhat more sensitive to oxidation in air than TPP. Nevertheless, exclusion of air is, in contrast to trialkylphosphines, not mandatory. Oxidation under typical reaction conditions (reaction time not longer than 24 h and reaction temperature below 80 °C) is slow and can be considered as unproblematic.

## Experimental

### General information

All experiments were performed under ambient conditions. Chemicals were purchased from Sigma-Aldrich, Carl Roth, Merck, or TCI and were used as received. The catalysts TPP and TMTPP were purchased from Sigma-Aldrich. MMTPP was prepared according to literature [42]. Stabilizers present in the Michael acceptors were not removed. ^1^H and ^13^C NMR spectra were recorded on a Bruker Avance 300 MHz spectrometer at 25 °C (^1^H: 300.36 MHz; ^13^C: 75.53 MHz). Chemical shifts δ are given in ppm relative to the residual protons and carbons of the deuterated solvent. (CHCl_3_: 7.26 ppm and 77.16 ppm, DMSO: 2.50 and 39.52 for ^1^H and ^13^C, respectively). ^31^P NMR measurements were performed on a Varian Inova 500 MHz instrument operating at 202.547 MHz. Chemical shifts are reported in ppm relative to an external standard (85% H_3_PO_4_). Spectra are ^1^H-decoupled and as delay time (d1) 25 s was set. Deuterated solvents were obtained from Cambridge Isotope Laboratories Inc. Size exclusion chromatography (SEC) was performed on a system provided by Shimadzu (equipped with two separating columns from MZ-Gel SD plus, 500 A and 100 A, linear 5 µ; UV detector (SPD-20A) and RI detector (RID-20A)) using THF as eluent. Poly(styrene) standards in the range of 350 to 17800 g/mol purchased from Polymer Standard Service were used for calibration.

### Computational details

All calculations were run with the TURBOMOLE program (version 7.4.1) [43]. Geometries were pre-optimized using the PBE [44] functional, the def2-SVPD [45–46] basis set and D3 [47] dispersion correction. All structures were then re-optimized using the hybrid functional B3LYP [48–51] D3 with the def2-TZVPPD basis set. For gas-phase calculations, temperature effects (298 K) and zero-point energies have been approximated by the rigid-rotor-harmonic oscillator (RRHO) approximation. The zero-point energies have been scaled by a factor of 1.0030 (B3LYP/def2-TZVPPD) and 1.0302 (PBE/def2-SVPD) to account for anharmonic effects [52]. Solvent effects of chloroform have been considered for calculation of the Gibbs free energy (Δ*G*) of zwitterion formation and were calculated by the conductor-like screening model (COSMO) [53–54] with a dielectric constant of 4.8 and a radius of 3.17. Our best estimate for the calculation of zwitterion energies resulted in using B3LYP-D3 /TZVPPD + Δsolv (B3LYP-D3) + ZPE,temp (PBE-D3/def2-SVPD).

### General procedure for oxa-Michael additions

The alcohol (2.0 equiv for mono-functionalized Michael acceptors, 3.0 equiv for **3**) and the catalyst (0.01 equiv) were added to a 4 mL-sealed tube. Then, the Michael acceptor was added, and the reaction mixture was stirred at room temperature or at 80 °C. The reaction progress was monitored by ^1^H NMR spectroscopy after 1 and 24 h. All experiments were performed at least three times.

### Oxa-Michael addition polymerization of 2-hydroxyethyl acrylate (**4**)

A 4 mL-glass tube was charged with phosphine (0.05 equiv) and **4** (1.0 equiv, 0.1 g, 0.861 mmol) and sealed. The reaction mixture was stirred at room temperature or at 80 °C. Samples taken after either 1 h or 24 h were evaluated by ^1^H NMR spectroscopy and SEC. All experiments were performed at least three times.

## Supporting Information

File 1 Experimental details, data in tabular form, NMR spectra.

File 2 xyz Files of the calculated structures.
