# Supplementary material for: Electron-rich triarylphosphines as nucleophilic catalysts for oxa-Michael reactions
Source: Beilstein J Org Chem. 2021 Jul 21;17:1689–97. doi: 10.3762/bjoc.17.117 (PMC8313974; doi:10.3762/bjoc.17.117)
Supplement: File 1 — Experimental details, data in tabular form, NMR spectra. [file Beilstein_J_Org_Chem-17-1689-s001.pdf]

BEILSTEIN JOURNAL OF ORGANIC CHEMISTRY

Supporting Information

for

Electron-rich triarylphosphines as nucleophilic catalysts for oxa-Michael reactions

Susanne M. Fischer, Simon Renner, A. Daniel Boese and Christian Slugovc

Beilstein J. Org. Chem. **2021,** *17,* 1689–1697. doi:10.3762/bjoc.17.117

Experimental details, data in tabular form, NMR spectra

License and Terms: This is a supporting information file under the terms of the Creative Commons Attribution License (https://creativecommons.org/licenses/by/4.0). Please note that the reuse, redistribution and reproduction in particular requires that the author(s) and source are credited and that individual graphics may be subject to special legal provisions.
The license is subject to the *Beilstein Journal of Organic Chemistry* terms and conditions: (https://www.beilstein-journals.org/bjoc/terms)

Experimental details

Synthesis of (4-methoxyphenyl)diphenylphosphine (MMTPP)

The synthesis was performed according to a literature procedure.[1] Under a nitrogen atmosphere, a 100 mL Schlenk tube was charged with dry THF (30 mL) and 4-bromoanisole (1.56 g, 8.34 mmol). The tube was cooled to −80 °C with an acetone–N_2 bath for 30 min before *n*-butyllithium (5.7 mL, 1.6 M in hexanes, 8.9 mmol, 1.1 equiv) was added. The reaction mixture was stirred for 1 h. Then, chlorodiphenylphosphine (2.51 g, 11.4 mmol, 1.33 equiv) dissolved in THF (6 mL) was added and the reaction mixture was stirred for another 4 h at −80 °C before it was warmed to room temperature overnight. The reaction was quenched with saturated NH_4Cl solution (15 mL) and water (15 mL). The organic phase was separated and subsequently washed with saturated NaCl solution (30 mL) and water (30 mL). The solvent was removed under reduced pressure whereupon a yellow, oily liquid remained. The crude product was purified by silica gel column chromatography with cyclohexane/dichloromethane 4:1 as eluent to give the product as colorless crystals (0.886 g, 3.03 mmol, 35.5% yield).

Reactions with acrylonitrile as Michael acceptor

Conversion of acrylonitrile was determined by integration of the signals deriving from the residual Michael acceptor (6.26–6.20 ppm, ^{1}H) and the CH_2 group in the β-position to the cyano group. NMR spectra of **1a–d** are exemplarily shown in Figure S1, Figure S2, Figure S3, and Figure S4.

Table S1: Conversion of acrylonitrile (%) in the oxa-Michael reaction with alcohols **a**–**d** after 1 h and 24 h catalyzed by 1 mol % phosphine at room temperature (23 °C).

	TPP		MMTPP		TMTPP	
Alcohol (Product)	1 h	24 h	1 h	24 h	1 h	24 h
Propan-2-ol (**1a**)	0	0	0	2.8	4.1	38.2
Propan-1-ol (**1b**)	2.4	26.7	16.3	65.9	73.2	97.6
Prop-2-en-1-ol (**1c**)	6.1	37.2	29.6	75.8	86.1	97.7
Prop-2-yn-1-ol (**1d**)	23.1	97.0	24.5	98.7	23.6	98.9

3-Isopropoxypropanenitrile[1] (1a):

Propan-2-ol (2.10 equiv, 0.238 g, 3.96 mmol), acrylonitrile (1.00 equiv, 124 µL, 1.89 mmol), tris(4-methoxyphenyl)phosphine (0.01 equiv, 0.00674 g, 0.0191 mmol).

^{1}H-NMR (300.36 MHz, $CDCl_3$): δ 3.66 – 3.61 (m, 3H, O-*CH*-$(CH_3)_2$, O-*CH_2*-CH_2), 2.57 (t, 2H, *CH_2*-CN), 1.20 (d, 6H, O-CH-*$(CH_3)_2$*).

3-Propoxypropanenitrile[2] (1b):

Propan-1-ol (2.10 equiv, 0.244 g, 4.06 mmol), acrylonitrile (1.00 equiv, 124 µL, 1.89 mmol), tris(4-methoxyphenyl)phosphine (0.01 equiv, 0.00652 g, 0.0185 mmol).

^{1}H-NMR (300.36 MHz, $CDCl_3$): δ 3.66 – 3.60 (m, 2H, O-*CH_2*-CH_2-CN), 3.44 (t, 2H, O-*CH_2*-CH_2), 2.59 (t, 2H, *CH_2*-CN), 1.60 (m, 2H, *CH_2*-CH_3), 0.93 (t, 3H, CH_2-*CH_3*).

3-(Allyloxy)propanenitrile[3] (1c):

Prop-2-en-1-ol (2.00 equiv, 0.219 g, 3.78 mmol), acrylonitrile (1.00 equiv, 124 µL, 1.89 mmol), tris(4-methoxyphenyl)phosphine (0.00668 g, 0.01 equiv, 0.0190 mol).

^{1}H-NMR (300.36 MHz, $CDCl_3$): δ 5.86-5.78 (m, 1H, CH_2=*CH*-CH_2), 5.27-5.13 (dd, 2H, *CH_2*=CH-CH_2), 3.98 (d, 2H, O-CH_2-CH), 3.60 (t, 2H, O-*CH_2*-CH_2), 2.55 (t, 2H, O-CH_2-*CH_2*)

3-(Prop-2-yn-1-yloxy)propanenitrile[4] (1d):

Prop-2-yn-1-ol (2.00 equiv, 0.223 g, 3.98 mmol), acrylonitrile (1.00 equiv, 124 µL, 1.89 mmol), tris(4-methoxyphenylphosphine (0.01 equiv, 0.00670 g, 0.0190 mmol).

^{1}H-NMR (300.36 MHz, $CDCl_3$): δ 4.22 (d, 2H, O-*CH_2*-C-), 3.76 (t, 2H, O-*CH_2*-CH_2), 2.65 (t, 2H, *CH_2*-CN), 2.48 (dt, 1H, *CH*-C-).

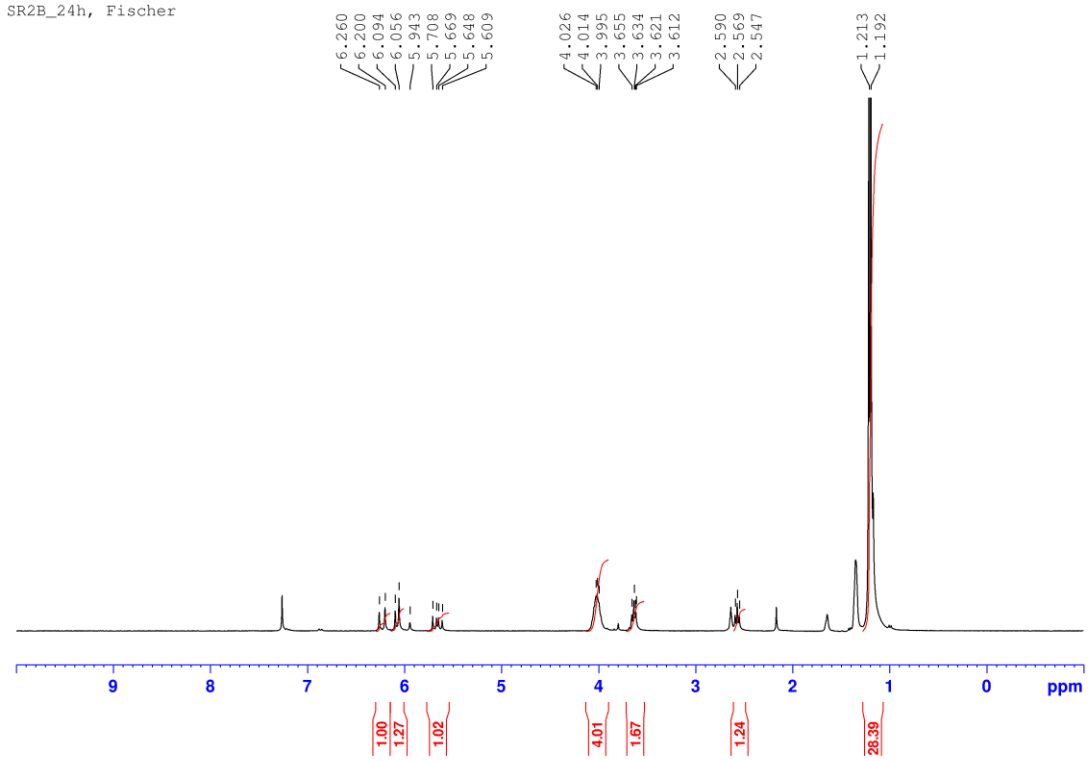

Figure S1: ^{1}H-NMR spectrum (300 MHz, $CDCl_3$) of **1a** after 24 h reaction time; as catalyst TMTPP was used; conversion was calculated from the integral ratio of the peaks at 6.26–6.20 and the peak at 2.57.

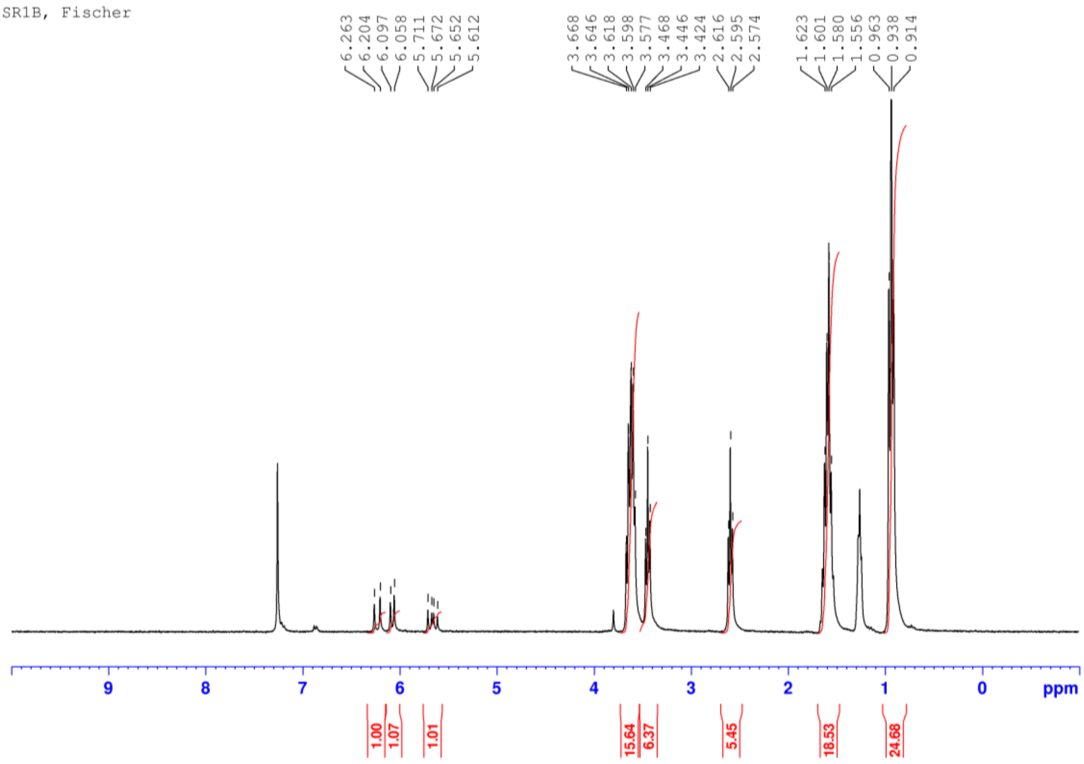

Figure S2: ^{1}H-NMR spectrum (300 MHz, $CDCl_3$) of **1b** after 1 h reaction time; as catalyst TMTPP was used; conversion was calculated from the integral ratio of the peaks at 6.26–6.20 and the peak at 2.59.

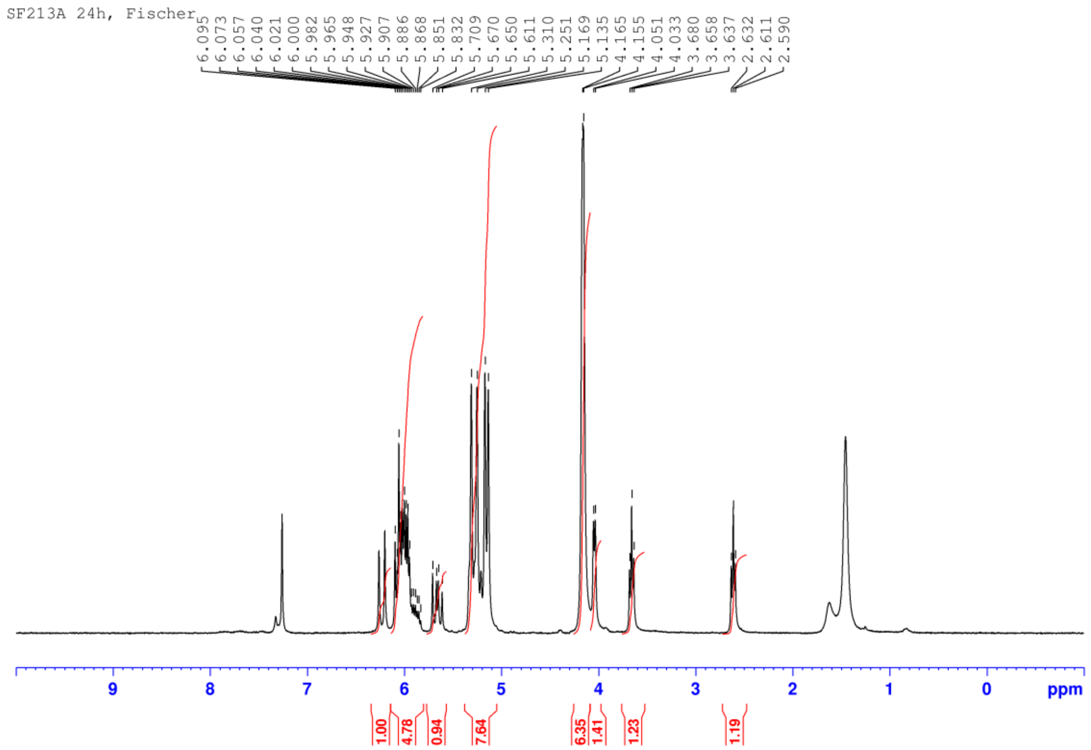

Figure S3: ^{1}H-NMR spectrum (300 MHz, $CDCl_3$) of **1c** after 24 h reaction time; as catalyst TPP was used; conversion was calculated from the integral ratio of the peaks at 6.26–6.20 and the peak at 2.61.

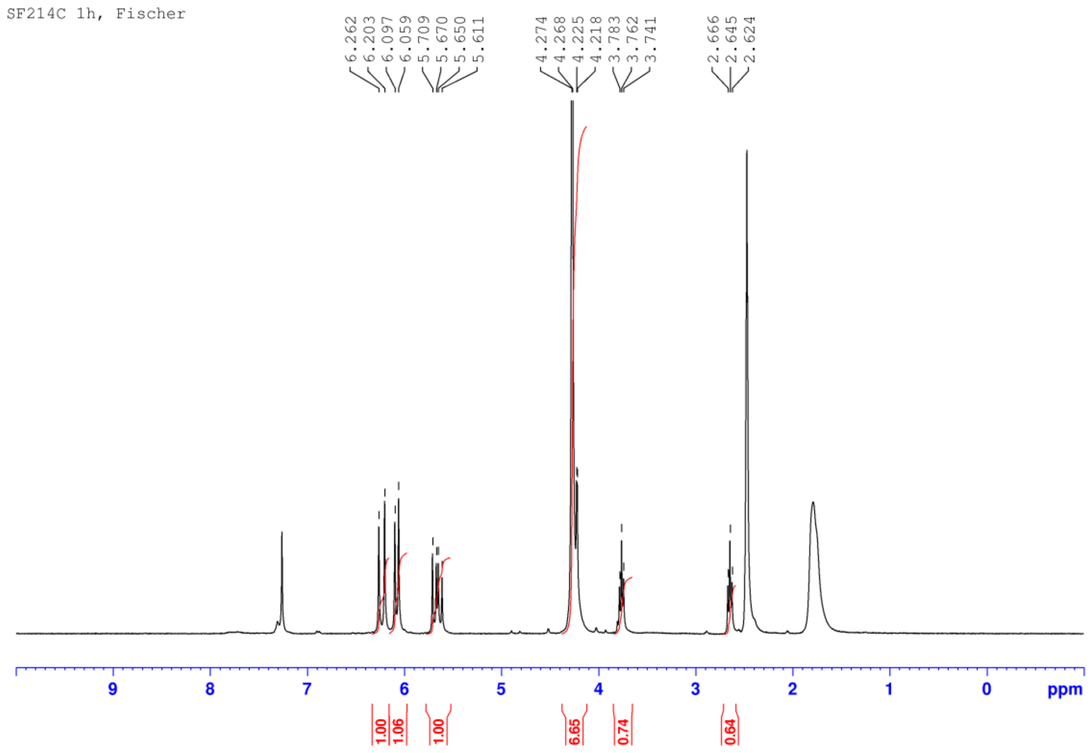

Figure S4: ^{1}H-NMR spectrum (300 MHz, $CDCl_3$) of **1d** after 1 h reaction time; as catalyst MMTPP was used; conversion was calculated from the integral ratio of the peaks at 6.26–6.20 and the peak at 2.65 ppm.

Reactions with acrylamide as Michael acceptor

Conversion of acrylamide was determined by integration of the signals deriving from the residual Michael acceptor (5.61–5.57 ppm, ^{1}H) and the newly formed CH_2 group. NMR spectra are exemplarily shown in Figure S5, Figure S6, Figure S7, and Figure S8.

Table S2: Conversion of acrylamide (%) in the oxa-Michael reaction with various alcohols after 1 h and 24 h catalyzed by 1 mol% phosphine at room temperature (23 °C)

	TPP		MMTPP		TMTPP	
Alcohol (Product)	1 h	24 h	1 h	24 h	1 h	24 h
Propan-2-ol (2**a**)	0	0	0	3.3	0	9.0
Propan-1-ol (2**b**)	0	7.6	0	19.0	3.6	60.6
Prop-2-en-1-ol (2**c**)	1.6	25.3	3.6	45.7	5.5	74.4
Prop-2-yn-1-ol (2**d**)	1.3	16.0	1.6	15.8	1.6	16.9

3-Isopropoxypropanamide (2a):

Propan-2-ol (2.00 equiv, 217 µL, 2.81 mmol), acrylamide (1.0 equiv, 0.101 g, 1.43 mmol), tris(4-methoxyphenyl)phosphine (0.01 equiv, 0.00498 g, 0.0141 mmol).

^{1}H-NMR (300 MHz, DMSO-d6) δ 7.26 (bs, 1H, NH_2), 6.76 (bs, 1H, NH_2), 3.55 (m, 3H, *CH*-O-*CH_2*), 2.23 (t, 2H, O=C-*CH_2*-CH_2), 1.05 (d, 6H, *CH_3*)

3-Propoxypropanamide (2b):

Propan-1-ol (2.00 equiv, 211 µL, 2.81 mmol), acrylamide (1.00 equiv, 0.0999 g, 1.40 mmol), tris(4-methoxyphenyl)phosphine (0.01 equiv, 0.00472 g, 0.0134 mmol).

^{1}H-NMR (300 MHz, DMSO-d6) δ 7.27 (bs, 1H, NH_2), 6.78 (bs, 1H, NH_2), 3.54 (t, 2H, O=C-CH_2-$\underline{CH_2}$), 3.31 (t, 2H,O-$\underline{CH_2}$-CH_2-CH_3), 1.47 (m, 2H, O-CH_2-$\underline{CH_2}$-CH_3), 0.84 (t, 3H, $\underline{CH_3}$)

^{13}C-NMR (75.53 MHz, DMSO-d6) δ 172.25 (C=O), 71.57 (CH_3-CH_2-$\underline{CH_2}$-O), 66.34 (O-$\underline{CH_2}$-CH_2-C=O), 35.89 (O-CH_2-$\underline{CH_2}$-C=O), 22.37 (CH3-$\underline{CH_2}$-CH_2-O), 10.48 (CH_3)

3-(Allyloxy)propenamide (2c):

Prop-2-en-1-ol (2.00 equiv, 191 µL, 2.81 mmol), acrylamide (1.00 equiv, 0.104 g, 1.46 mmol), tris(4-methoxyphenyl)phosphine (0.01 equiv, 0.00500 g, 0.0141 mmol).

^{1}H-NMR (300 MHz, DMSO-d6) δ 7.29 (bs, 1H, NH_2), 6.79 (bs, 1H, NH_2), 5.92-5.79 (m, 1H, CH_2=$\underline{CH}$-CH_2), 5.27-5.11 (dd, 2H, $\underline{CH_2}$=CH-CH_2), 3.91 (d, 2H, CH_2=CH-$\underline{CH_2}$), 3.56 (t, 2H, O-$\underline{CH_2}$-CH_2), 2.29 (t, 2H, O-CH_2-$\underline{CH_2}$)

^{13}C-NMR (75.53 MHz, DMSO-d6) δ 172.17 (C=O), 135.27 (CH_2=$\underline{CH}$-CH_2), 116.20 ($\underline{CH_2}$=CH-CH_2), 70.78 (CH_2=CH-$\underline{CH_2}$), 66.01 (O-$\underline{CH_2}$-CH_2), 35.81 (O-CH_2-$\underline{CH_2}$)

3-(Prop-2-yn-1-yloxy)propenamide (2d):

Prop-2-yn-1-ol (2.00 equiv, 166 µL, 2.81 mmol), acrylamide (1.00 equiv, 0.101 g, 1.42 mmol), tris(4-methoxyphenyl)phosphine (0.01 equiv, 0.00509 g, 0.0144 mmol).

^{1}H-NMR (300 MHz, DMSO-d6) δ 7.31 (bs, 1H, NH_2), 6.82 (bs, 1H, NH_2), 4.09 (d, 2H, CH-C-$\underline{CH_2}$), 3.62 (t, 2H, O-$\underline{CH_2}$-CH_2), 3.39 (t, 1H, $\underline{CH}$-C-CH_2), 2.29 (t, 2H, O-CH_2-$\underline{CH_2}$)

^{13}C-NMR (75.53 MHz, DMSO-d6) δ 172.06 (C=O), 80.34 (CH-$\underline{C}$-CH_2), 76.98 ($\underline{CH}$-C-CH_2), 65.69 (O-$\underline{CH_2}$-CH2), 57.32 (CH-C-$\underline{CH_2}$), 35.51 (O-CH_2-$\underline{CH_2}$)

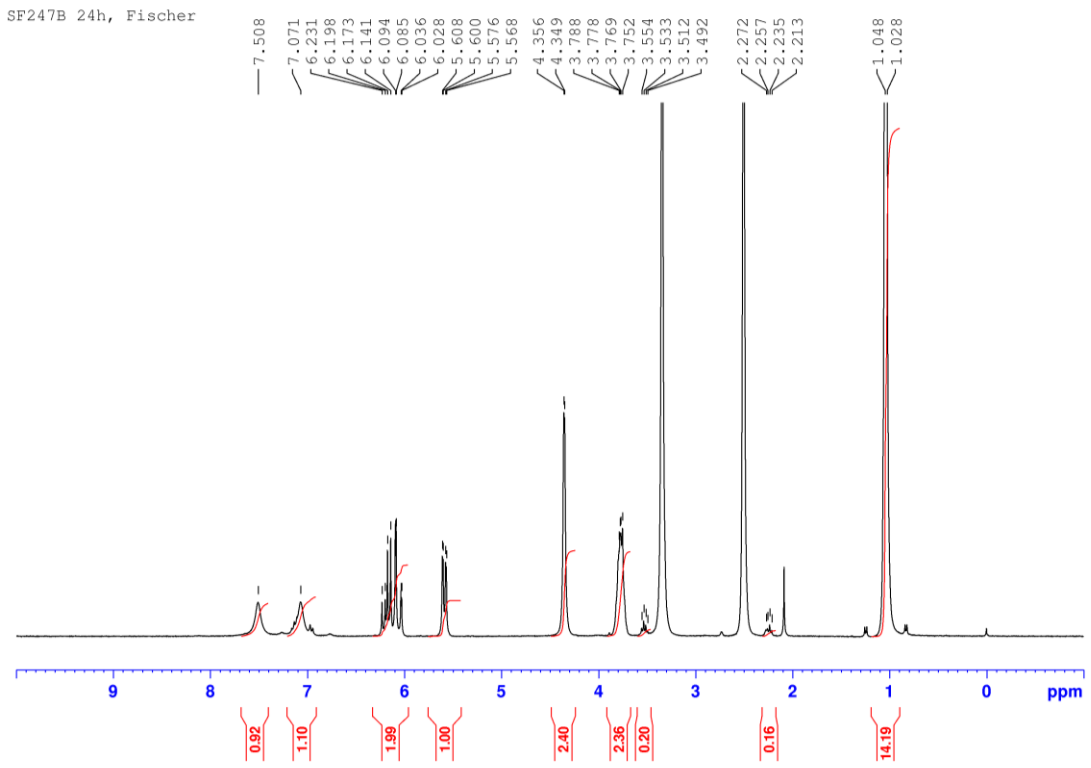

Figure S5: ^{1}H-NMR spectrum (300 MHz, DMSO-d_6) of **2a** after 24 h reaction time; as catalyst TMTPP was used; conversion was calculated from the integral ratio of the peaks at 5.61–5.58 and the peak at 3.53.

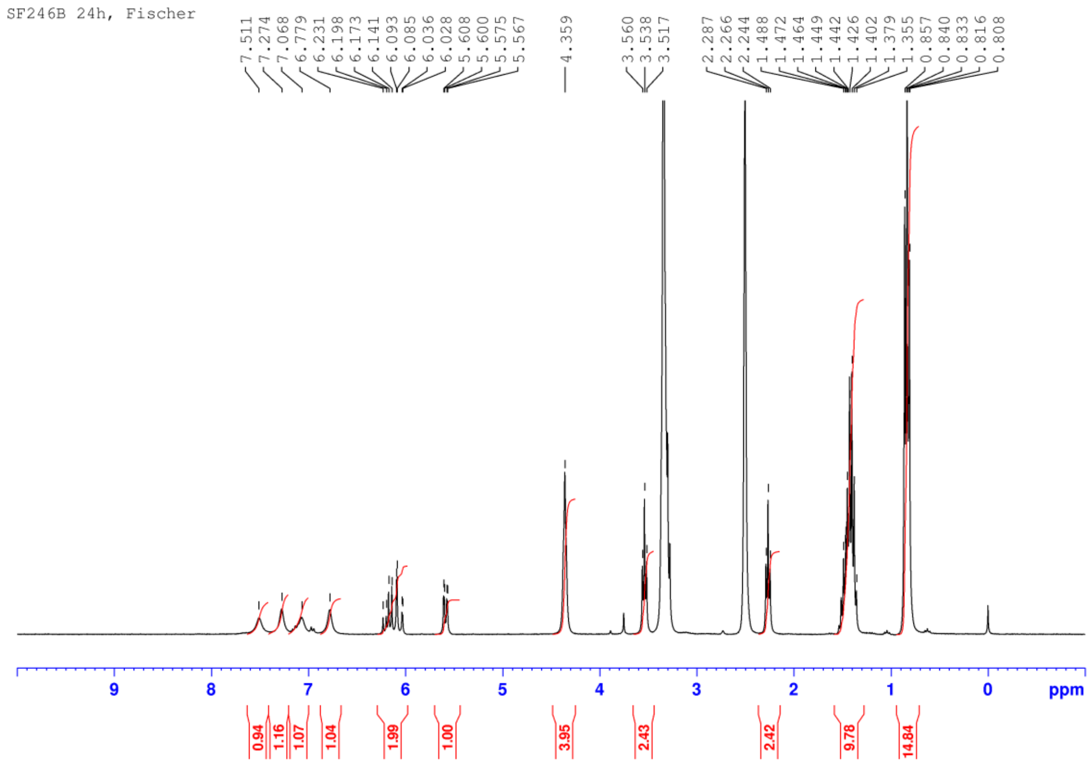

Figure S6: ^{1}H-NMR spectrum (300 MHz, DMSO-d_6) of **2b** after 24 h reaction time; as catalyst TMTPP was used; conversion was calculated from the integral ratio of the peaks at 5.61–5.58 and the peak at 3.54.

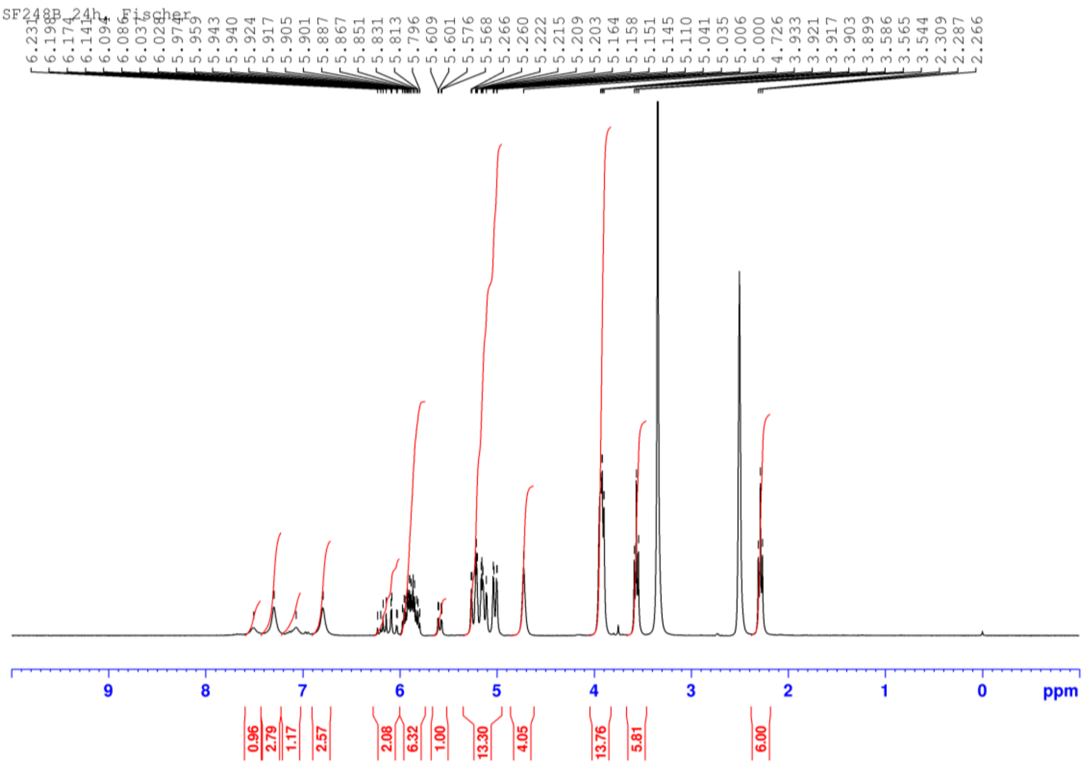

Figure S7: ^{1}H-NMR spectrum (300 MHz, DMSO-d_6) of **2c** after 24 h reaction time; as catalyst TMTPP was used; conversion was calculated from the integral ratio of the peaks at 5.61–5.58 and the peak at 3.56.

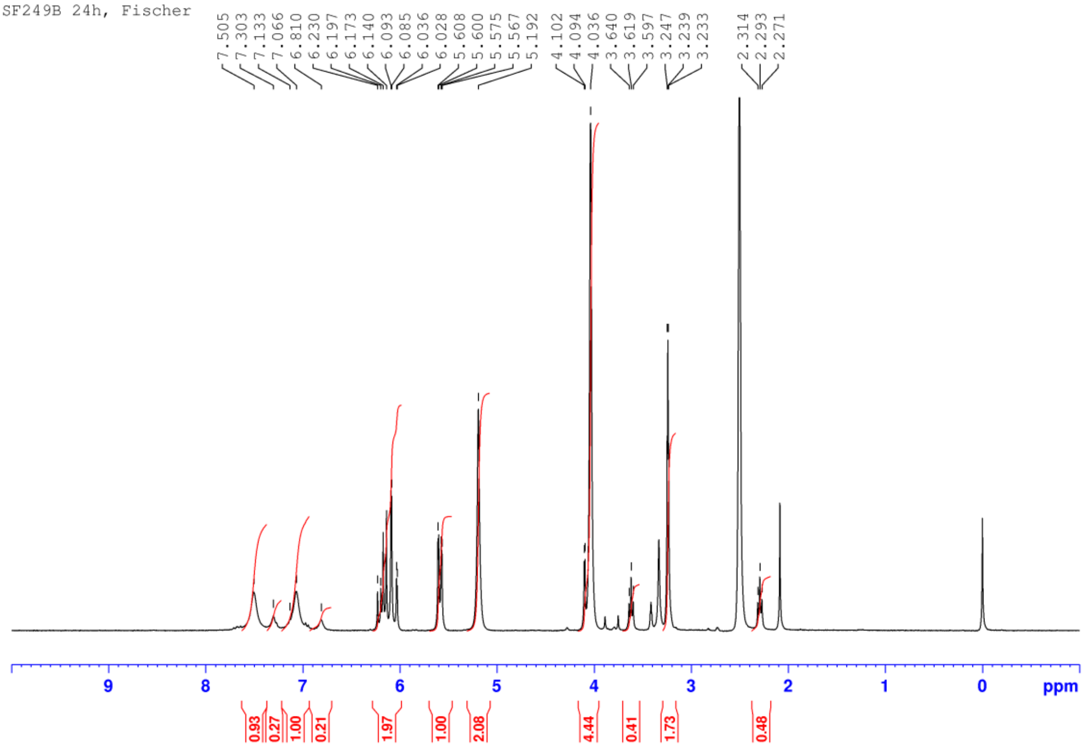

Figure S8: ^{1}H-NMR spectrum (300 MHz, DMSO-d_6) of **2d** after 24 h reaction time; as catalyst TMTPP was used; conversion was calculated from the integral ratio of the peaks at 5.61–5.58 and the peak at 3.61.

Reactions with divinyl sulfone as Michael acceptor

Conversion of divinyl sulfone was determined by integration of the signals deriving from the residual Michael acceptor (6.42–6.37 ppm, 1H) and the CH_2 group in β-position. NMR spectra are exemplarily shown in Figure S9, Figure S10, Figure S11, and Figure S12.

Mono/di ratios were determined from the integral ratio of the signals deriving from the protons adjacent to the sulfur atom (details see figures).

Table S3: Conversion of divinyl sulfone and corresponding product ratios mono/di [%] in the oxa-Michael reaction with alcohols **a–d** after 1 h and 24 h catalyzed by 1 mol % phosphine at room temperature (23 °C)

	TPP		MMTPP		TMTPP	
Alcohol (Product)	1 h	24 h	1 h	24 h	1 h	24 h
Propan-2-ol (**3a**)	53/3	52/48	58/3	33/67	65/5	17/83
Propan-1-ol (**3b**)	28/72	0/100	28/72	0/100	30/70	0/100
Prop-2-en-1-ol (**3c**)	29/70	0/100	42/58	11/89	21/79	0/100
Prop-2-yn-1-ol (**3d**)	40/60	0/100	52/48	0/100	31/69	0/100

2-(2-((2-Isopropoxyethyl)sulfonyl)ethoxy)propane (3a):

Propan-2-ol (3.00 equiv, 194 µL, 2.54 mmol), divinyl sulfone (1.00 equiv, 85.1 µL, 0.847 mmol), tris(4-methoxyphenyl)phosphine (0.01 equiv, 0.00305 g, 0.00866 mmol)

^{1}H-NMR (300 MHz, $CDCl_3$) δ 3.84 (t, 4H, C$\underline{H_2}$-S-C$\underline{H_2}$, 3.63 (m, 2H, $\underline{CH}$), 3.31 (t, 4H, -CH_2-$\underline{CH_2}$-S-), 1.16 (d, 12H, $\underline{CH_3}$).

1-(2-((2-Propoxyethyl)sulfonyl)ethoxy)propane (3b):

Propan-1-ol (3.00 equiv, 191 µL, 2.54 mmol), divinyl sulfone (1.00 equiv, 85.1 µL, 0.847 mmol), tris(4-methoxyphenyl)phosphine (0.01 equiv, 0.00292 g, 0.00828 mmol)

^{1}H-NMR (300 MHz, $CDCl_3$) δ 3.85 (dd, 4H, -$\underline{CH_2}$-CH_2-S-), 3.42 (t, 4H, CH_3-CH_2-$\underline{CH_2}$-), 3.32 (t, 4H, -CH_2-$\underline{CH_2}$-S-), 1.70-1.43 (m, 4H, CH_3-$\underline{CH_2}$-), 0.91 (t, 6H, $\underline{CH_3}$-).

^{13}C-NMR (75.53 MHz, $CDCl_3$) δ 73.15 (CH_3-CH_2-$\underline{CH_2}$-), 64.30 (-CH_2-$\underline{CH_2}$-S-), 55.03 (-$\underline{CH_2}$-CH_2-S-), 22.81 (CH_3-$\underline{CH_2}$-), 10.65 (CH_3-)

3-(2-((2-(Allyloxy)ethyl)sulfonyl)ethoxy)prop-1-ene (3c):

Prop-2-en-1-ol (3.00 equiv, 174 µL, 2.54 mmol), divinyl sulfone (1.0 equiv, 85.1 µL, 0.847 mmol), tris(4-methoxyphenyl)phosphine (0.01 equiv, 0.00298 g, 0.00846 mmol)

^{1}H-NMR (300 MHz, $CDCl_3$) δ 5.91 – 5.82 (m, 2H, CH_2-$\underline{CH}$-), 5.22 (m, 4H, $\underline{CH_2}$-CH-), 4.03 (d, 4H, -CH-$\underline{CH_2}$-O-), 3.88 (t, 4H, -$\underline{CH_2}$-CH_2-S-), 3.36 (t, 4H, -CH_2-$\underline{CH_2}$-S-).

3-(2-((2-(Prop-2-yn-1-yloxy)ethyl)sulfonyl)ethoxy)prop-1-yne (3d):

Prop-2-yn-1-ol (3.0 equiv, 152.2 µL, 2.54 mmol), divinyl sulfone (1.0 equiv, 85.1 µL, 0.847 mmol), tris(4-methoxyphenyl)phosphine (0.01 equiv, 0.00290 g, 0.00823 mmol)

^{1}H-NMR (300 MHz, $CDCl_3$) δ 4.27 (d, 4H, -C-$\underline{CH_2}$-), 3.98 (t, 4H, -$\underline{CH_2}$-CH_2-S-), 3.36 (t, 4H, -CH_2-$\underline{CH_2}$-S-), 2.48 (d, 2H, $\underline{CH}$-C-)

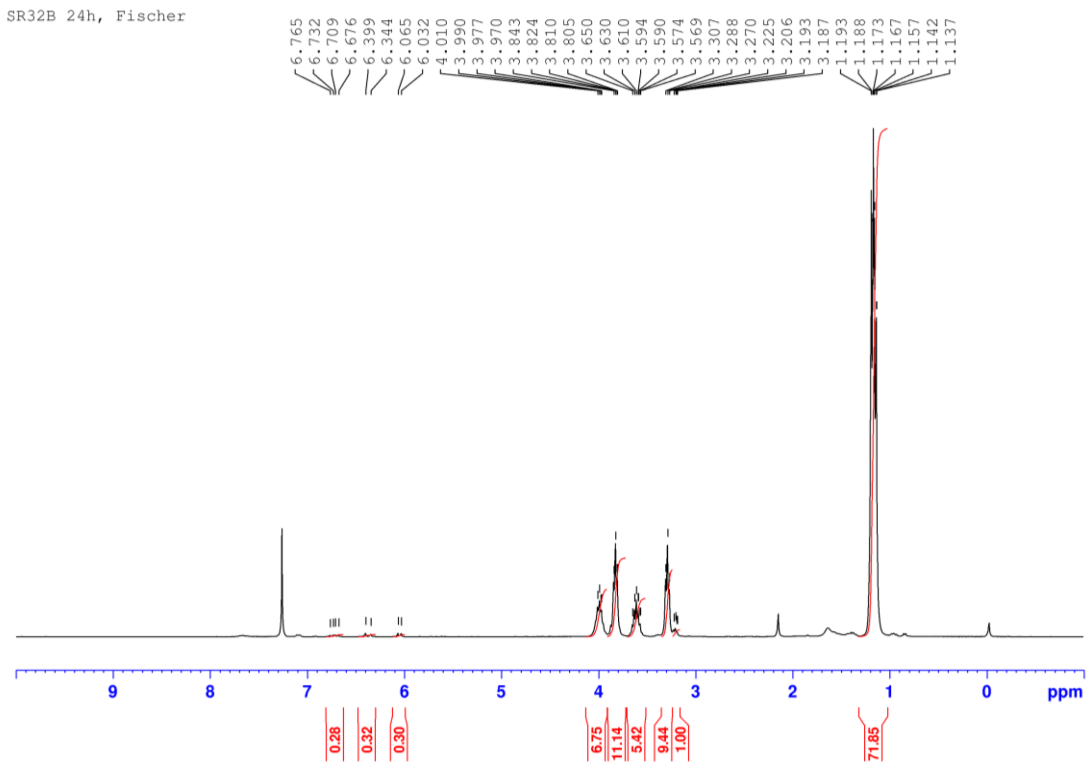

Figure S9: ^{1}H-NMR spectrum (300 MHz, $CDCl_3$) of **3a** after 24 h reaction time; as catalyst TMTPP was used; mono/di ratio was calculated from the peak at 3.19 and the peak at 3.29 ppm.

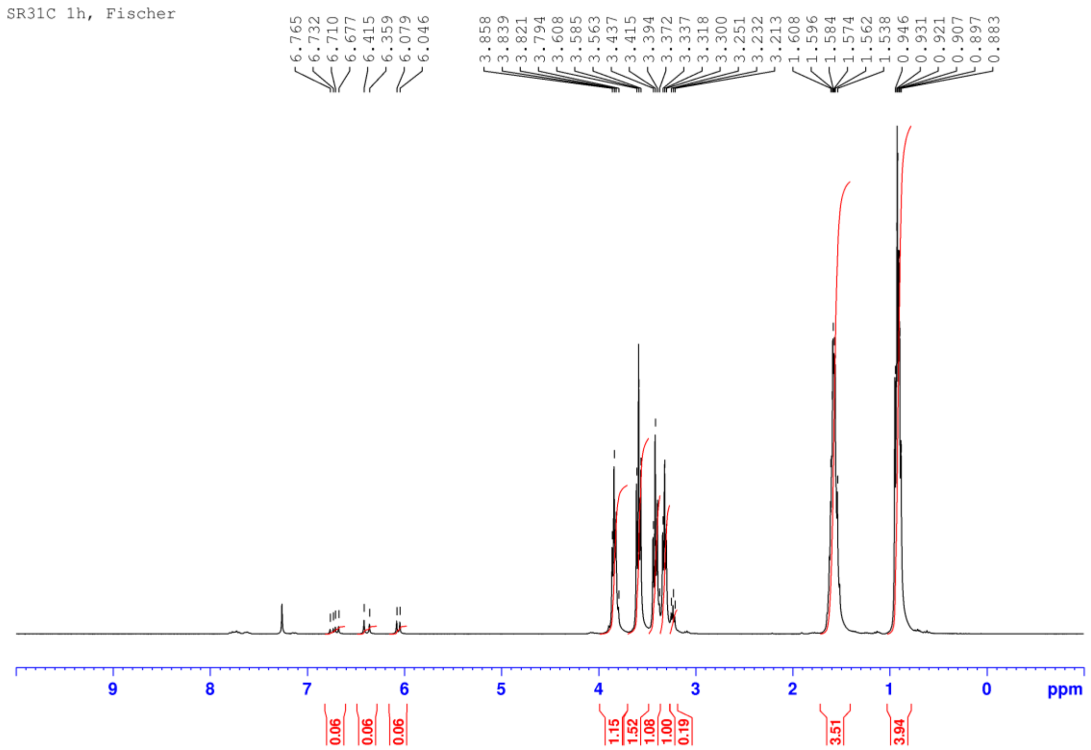

Figure S10: ^{1}H-NMR spectrum (300 MHz, $CDCl_3$) of **3b** after 1 h reaction time; as catalyst MMTPP was used; mono/di ratio was calculated from the peak at 3.23 and the peak at 3.32 ppm.

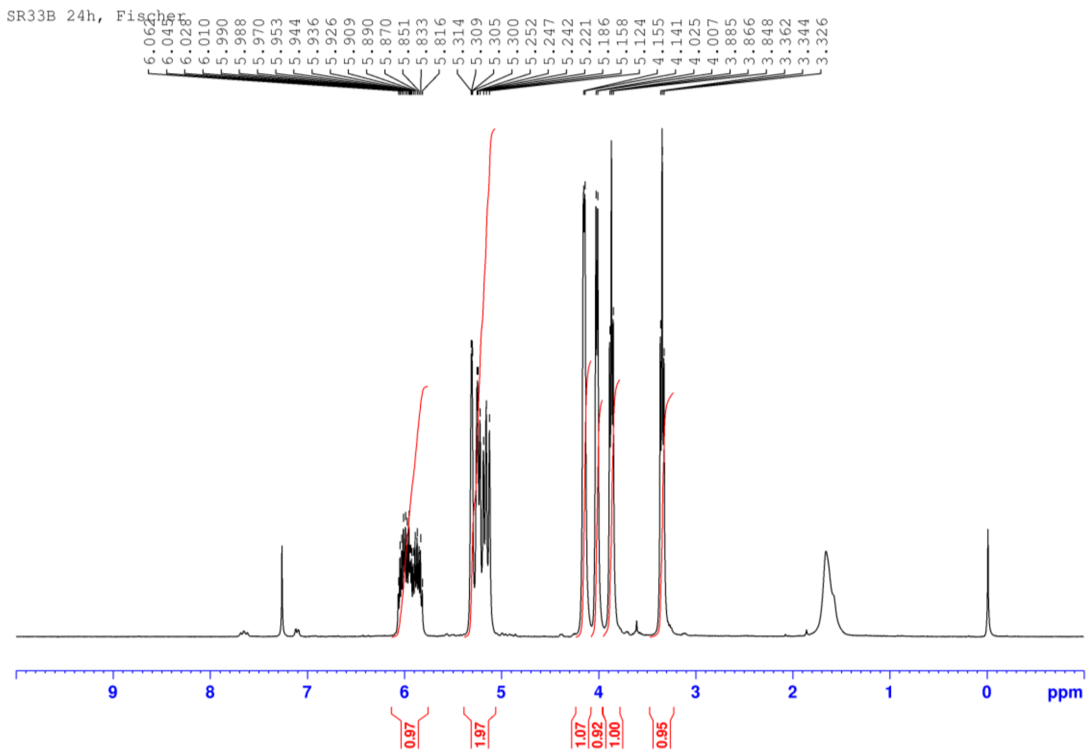

Figure S11: ^{1}H-NMR spectrum (300 MHz, $CDCl_3$) of **3c** after 24 h reaction time; as catalyst TMTPP was used; mono/di ratio was calculated from the peak at 3.26 (not visible in this spectrum) and the peak at 3.35 ppm.

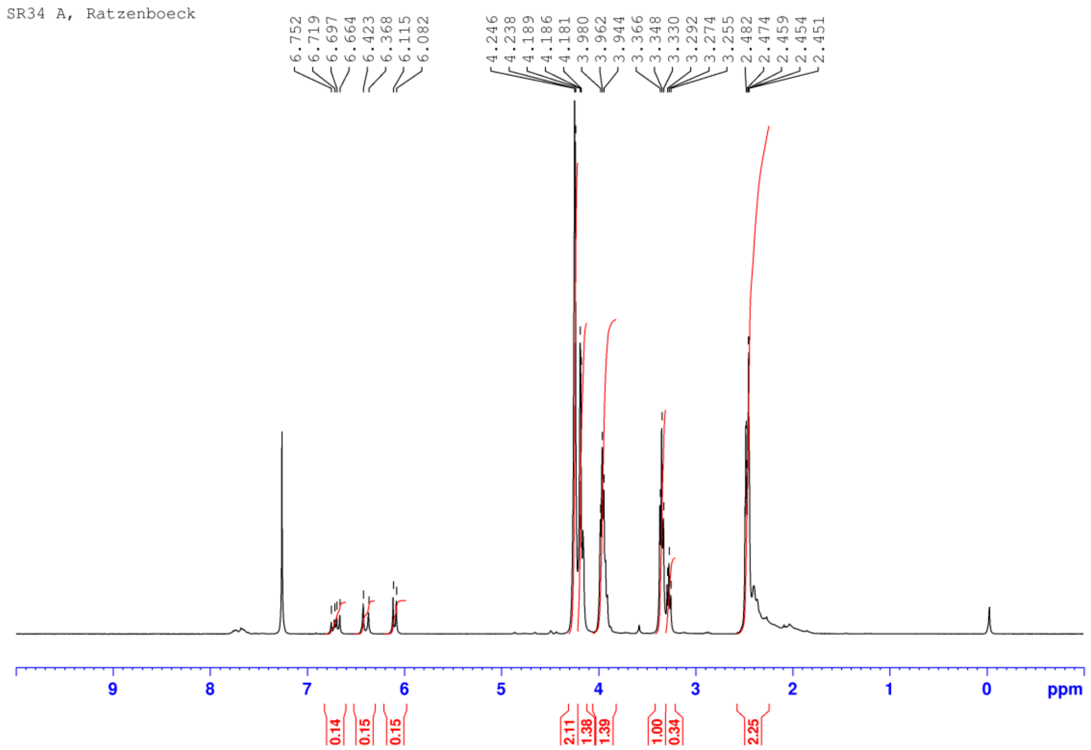

Figure S12: ^{1}H-NMR spectrum (300 MHz, $CDCl_3$) of **3d** after 24 h reaction time; as catalyst TPP was used; mono/di ratio was calculated from the peak at 3.27 and the peak at 3.35 ppm.

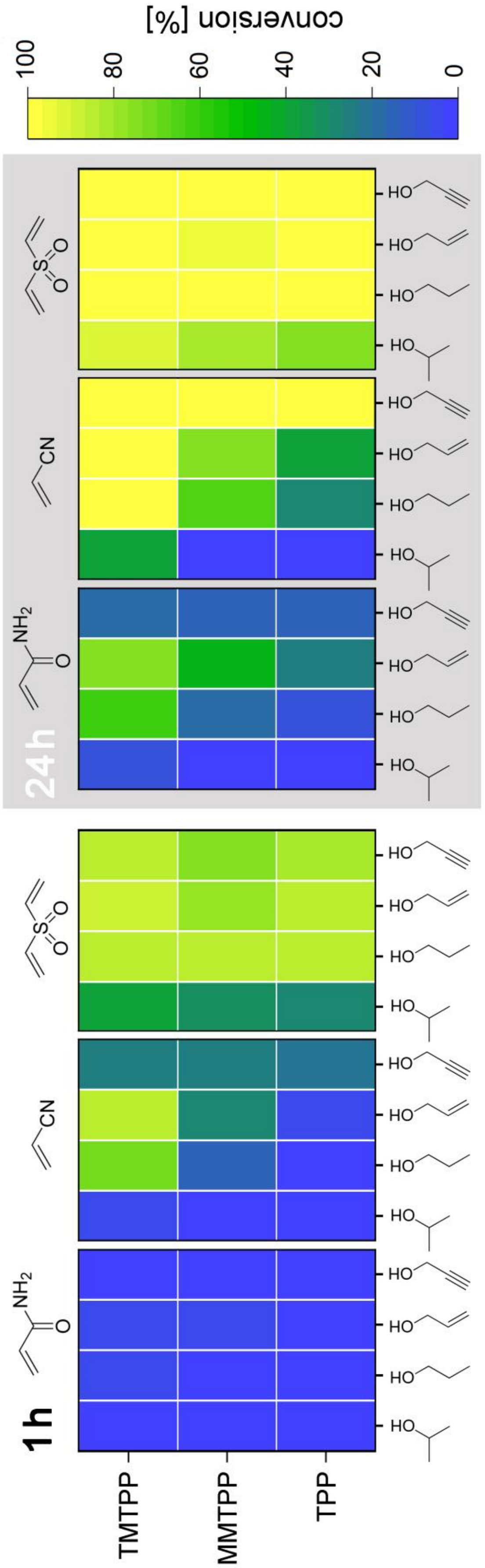

Figure S13: Heat-map plot of all reactions

Oxa-Michael addition polymerization of 2-hydroxyethyl acrylate (HEA)

Calculation of double bond conversion of 2-hydroxyethyl acrylate:

$$Double\ bond\ conversion\ [\%] = \frac{integral\ 2.62\ ppm\ /2}{integral\ 5.85 + integral\ 2.62/2} * 100$$

Calculation of Rauhut–Currier share:

$$Rauhut - Currier\ share\ [\%] = \frac{integral\ 5.61}{\frac{integral\ 2.62 - 4 * integral\ 5.61}{2}} * 100$$

Table S4: Conversion of 2-hydroxyethyl acrylate [%] after 24 h, molecular weight M_n of the formed polymers, polydispersity index (D) and Rauhut–Currier share [%]

Catalyst	T [°C]	conversion [%]	M_n [g/mol]	Đ	Rauhut-Currier share [%]
TPP	23	74	660	1.5	5
TPP	80	89	680	1.6	20
MMTPP	23	87	910	1.7	6
MMTPP	80	97	820	1.7	17
TMTPP	23	95	1160	1.8	6.5
TMTPP	80	99	890	1.8	17

^{1}H-NMR (300 MHz, $CDCl_3$) δ 6.47 (d, 1H), 6.20 (s, 6H), 5.84 (d, 1H), 4.33-4.21 (m, 76 H), 3.82-3.56 (m, 141 H), 2.61 (t, 70H).

^{13}C-APT NMR (75.53 MHz, $CDCl_3$) δ 171.4, 126.6, 72.27, 70.39, 68.96, 66.80-66.15, 64.07, 63.88, 63.62, 62.33, 61.66, 60.98, 34.99.

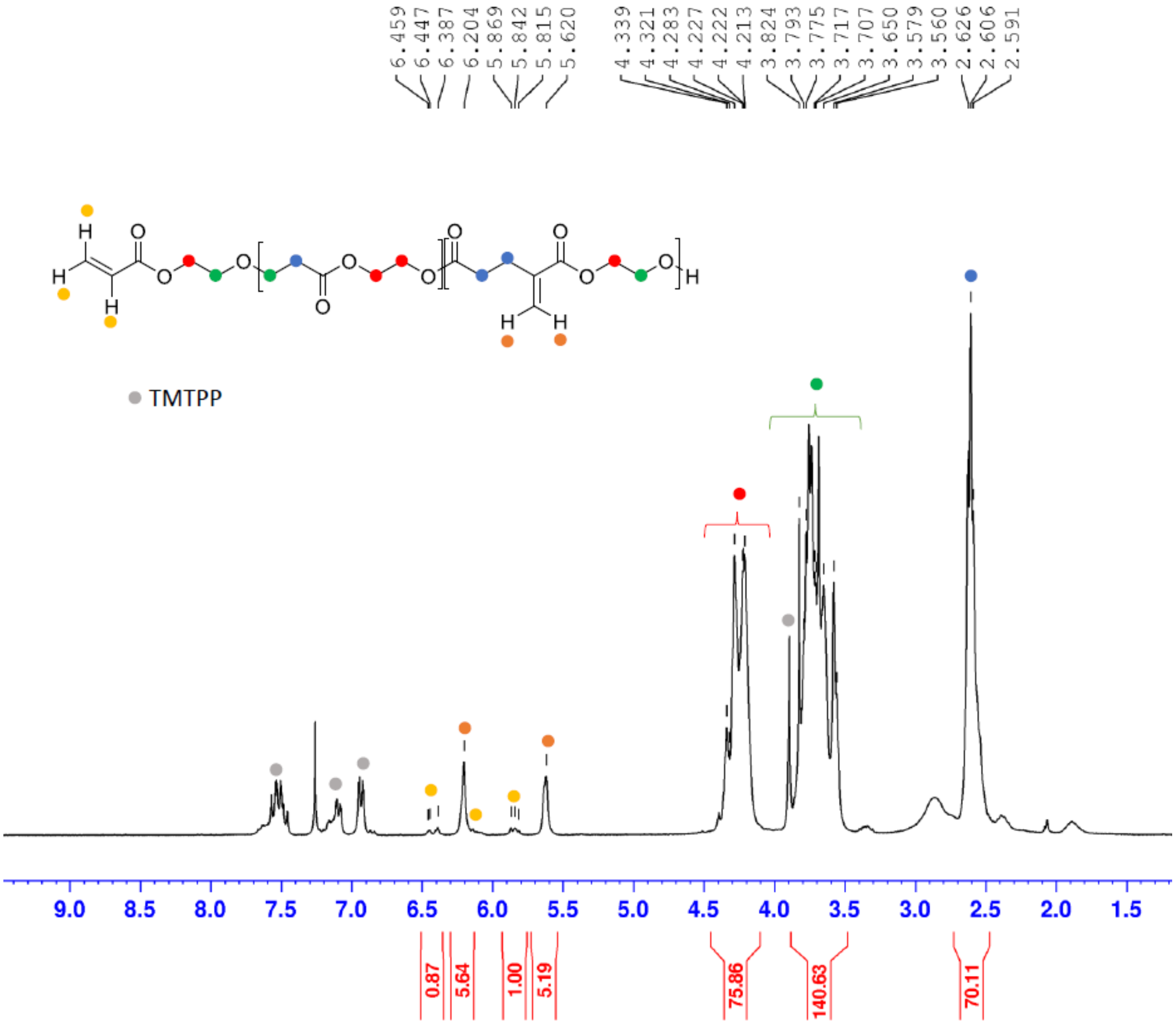

Figure S14: ^{1}H-NMR spectrum (300 MHz, $CDCl_3$) of poly4 after 24 h at 80°C with TMTPP (5 mol %) as catalyst

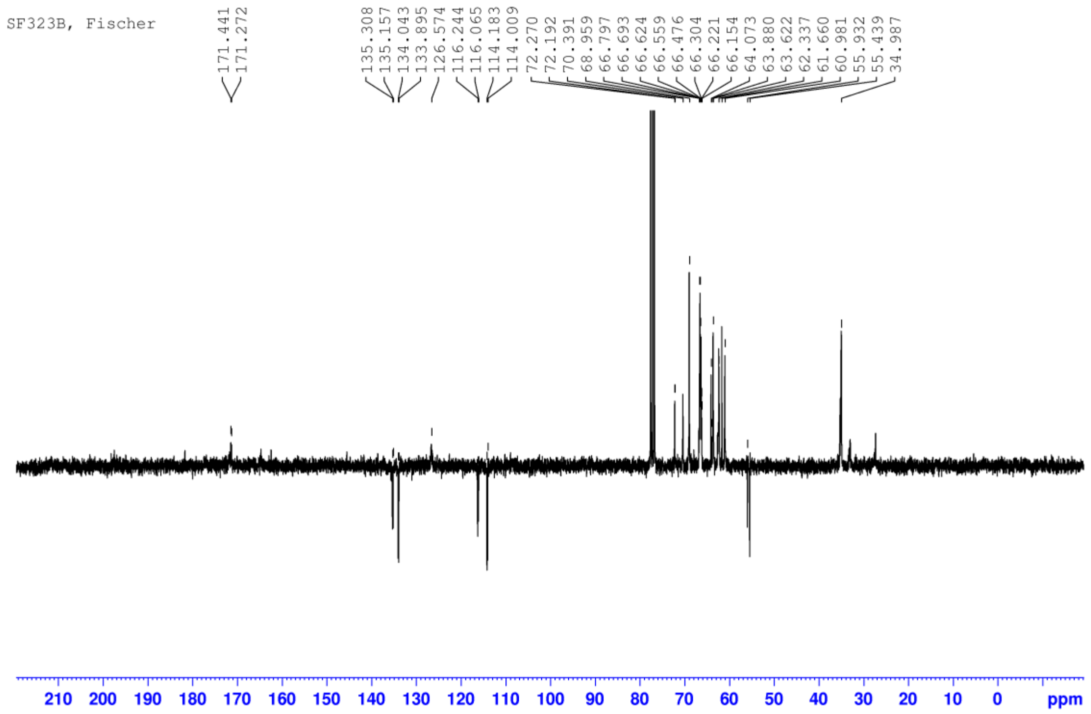

Figure S15: ^{13}C-APT spectrum (300 MHz, $CDCl_3$) of poly4 after 24 h at 80 °C with TMTPP (5 mol %) as catalyst

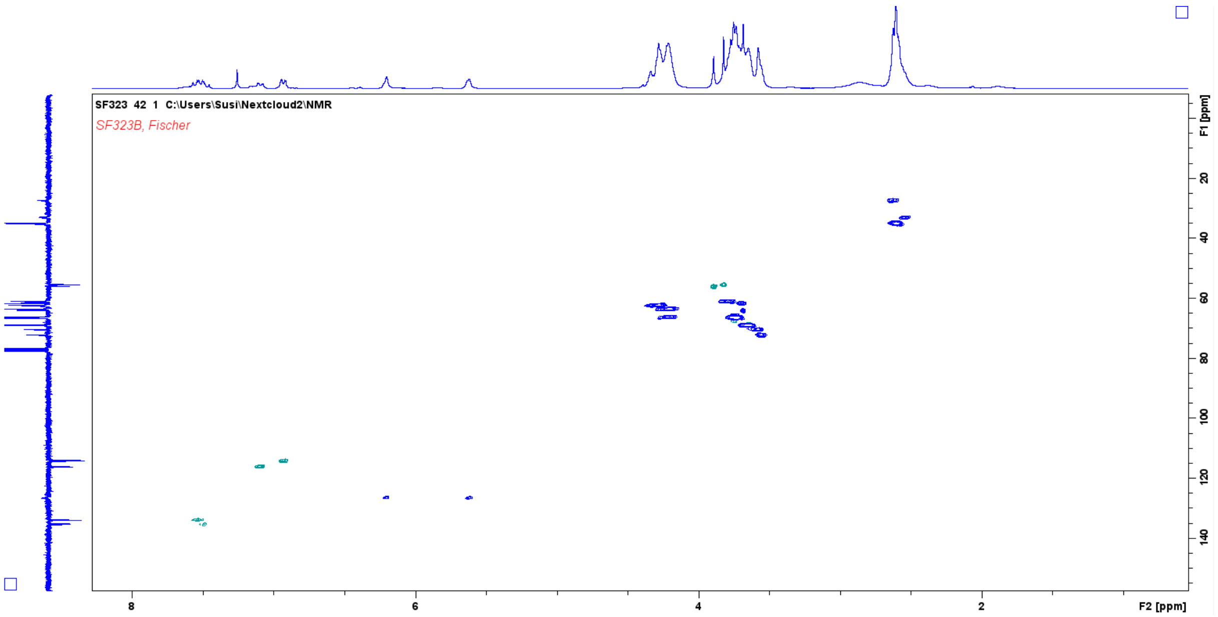

Figure S16: HSQC of poly4 after 24 h at 80 °C with TMTPP (5 mol %) as catalyst

Oxidation experiments (^{31}P-NMR)

Table S5: ^{31}P-NMR shifts (in $CDCl_3$) of TPP, MMTPP and TMTPP and their corresponding phosphine oxides relative to 85% H_3PO_4

	TPP	MMTPP	TMTPP
phosphine	-5.02	-6.69	-9.82
phosphine oxide	29.42	29.24	28.96

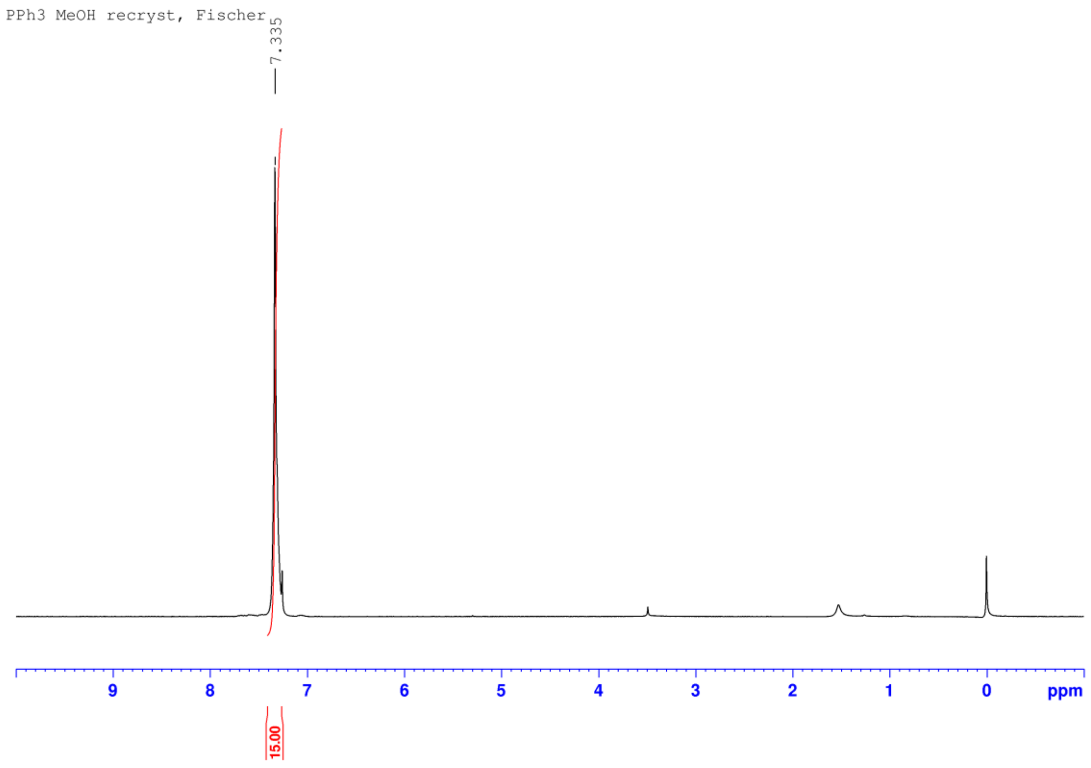

Figure S17: ^{1}H-NMR (300 MHz, $CDCl_3$) spectrum of TPP

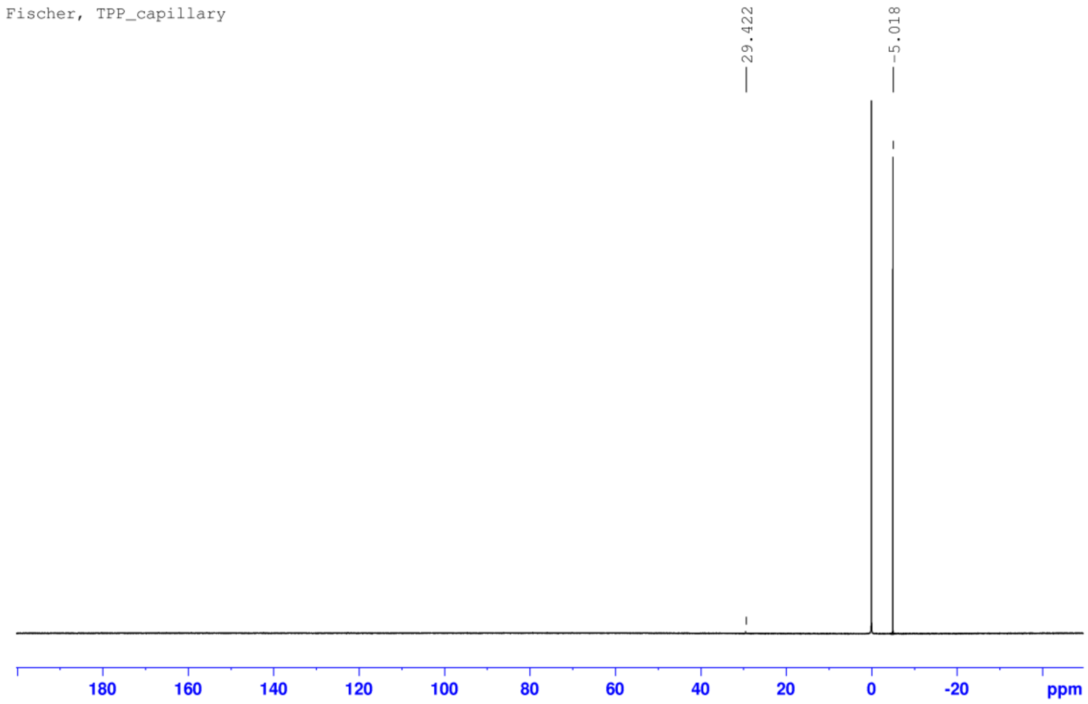

Figure S18: ^{31}P-NMR (202.55 MHz, $CDCl_3$) spectrum of TPP

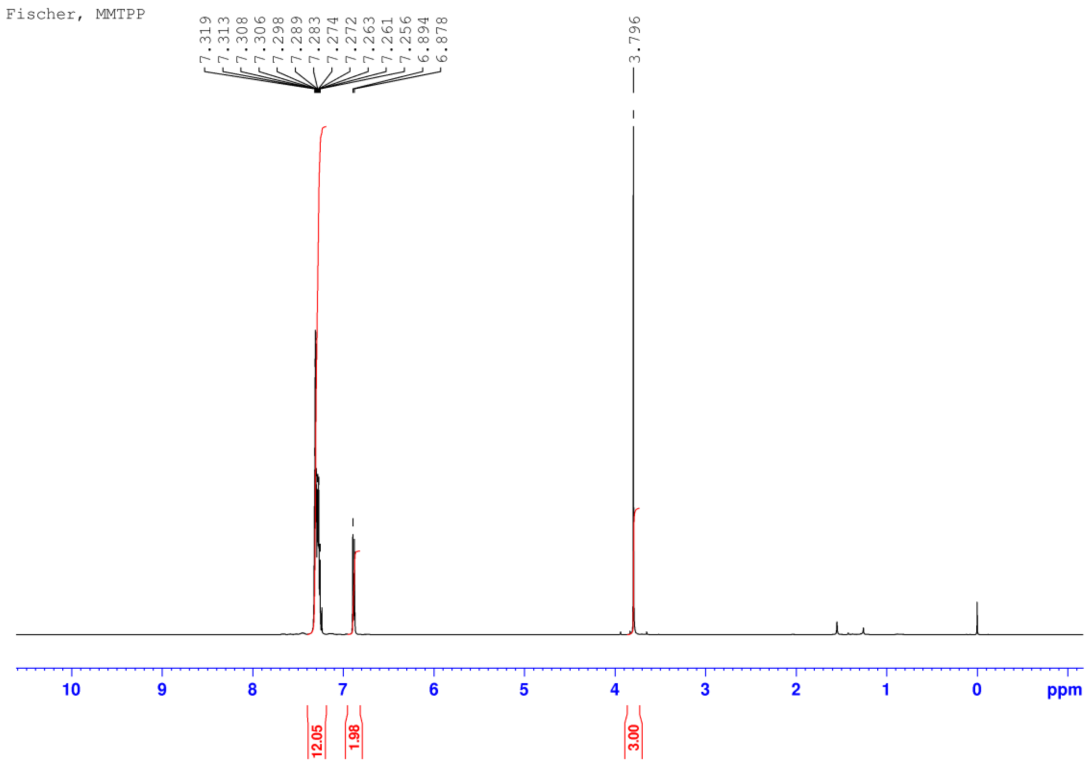

Figure S19: ^{1}H-NMR (500 MHz, $CDCl_3$) spectrum of MMTPP

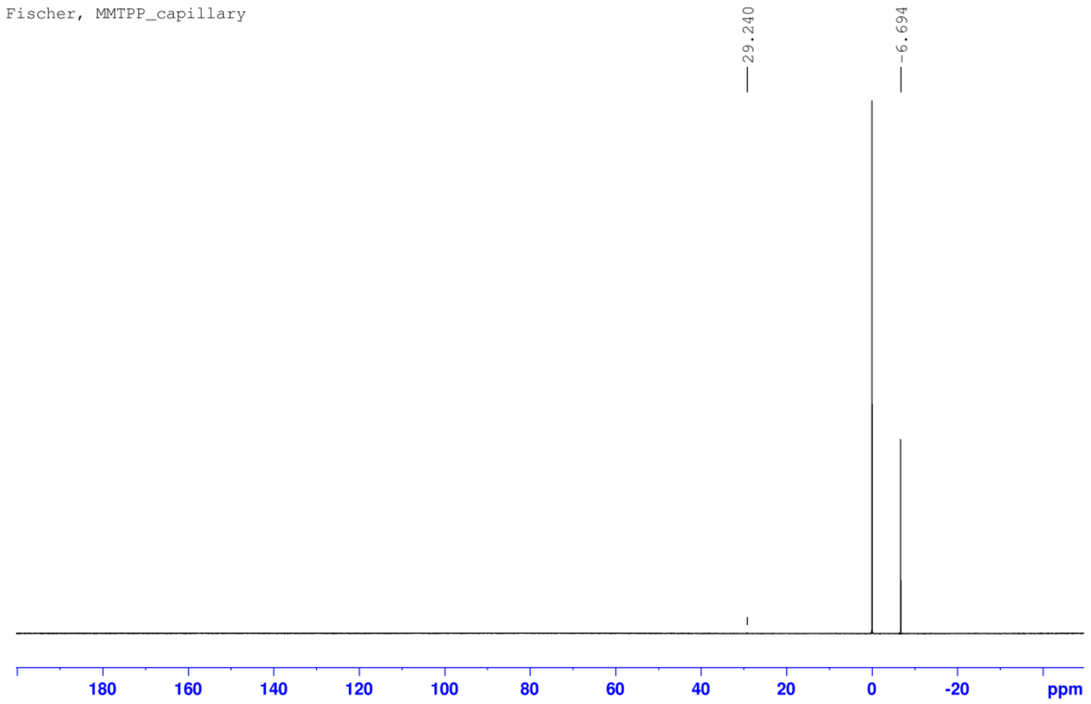

Figure S20: ^{31}P-NMR (202.55 MHz, $CDCl_3$) spectrum of MMTPP

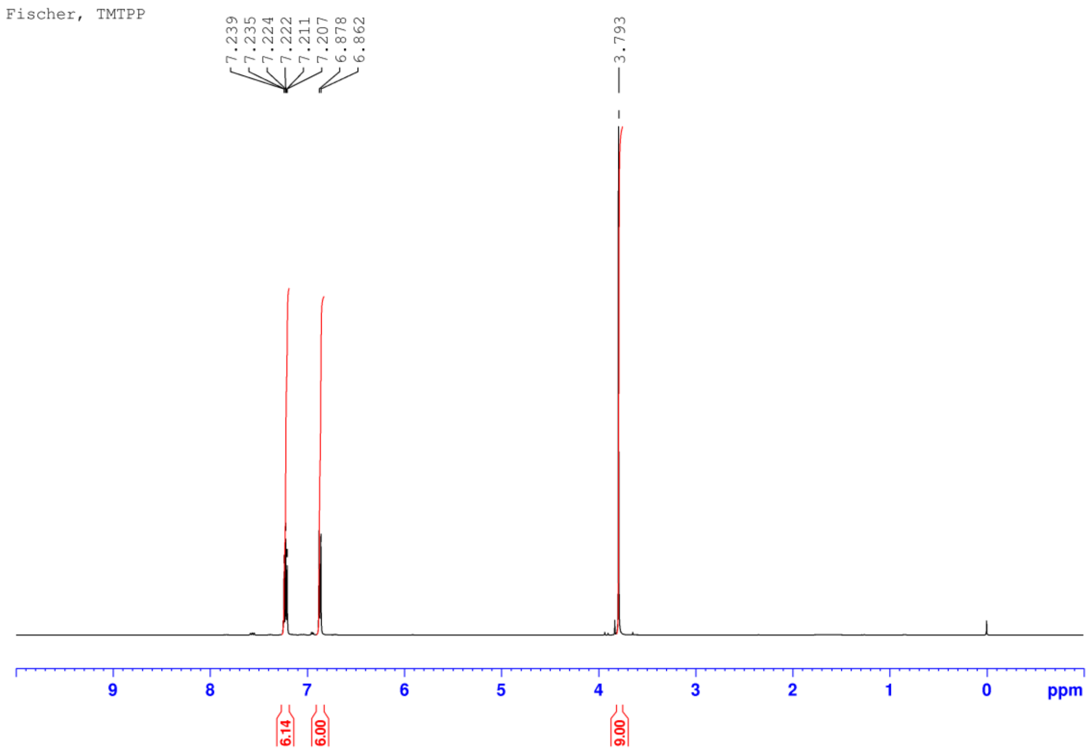

Figure S21: ^{1}H-NMR (500 MHz, $CDCl_3$) spectrum of TMTPP

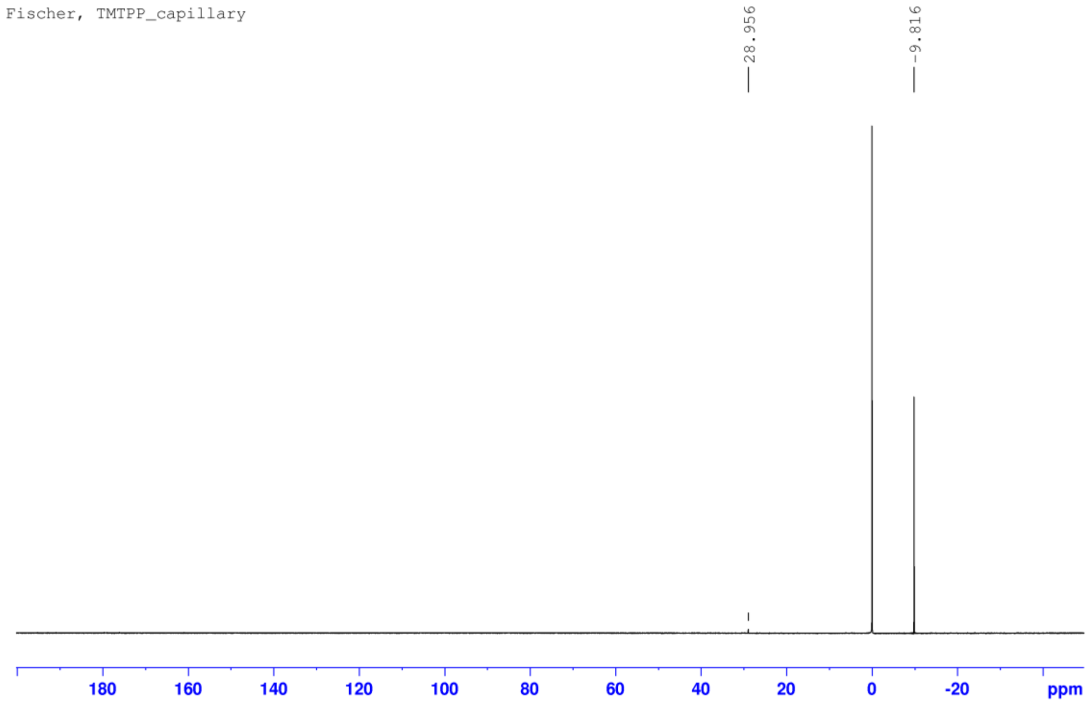

Figure S22: ^{31}P-NMR (202.55 MHz, $CDCl_3$) spectrum of TMTPP

Computational details

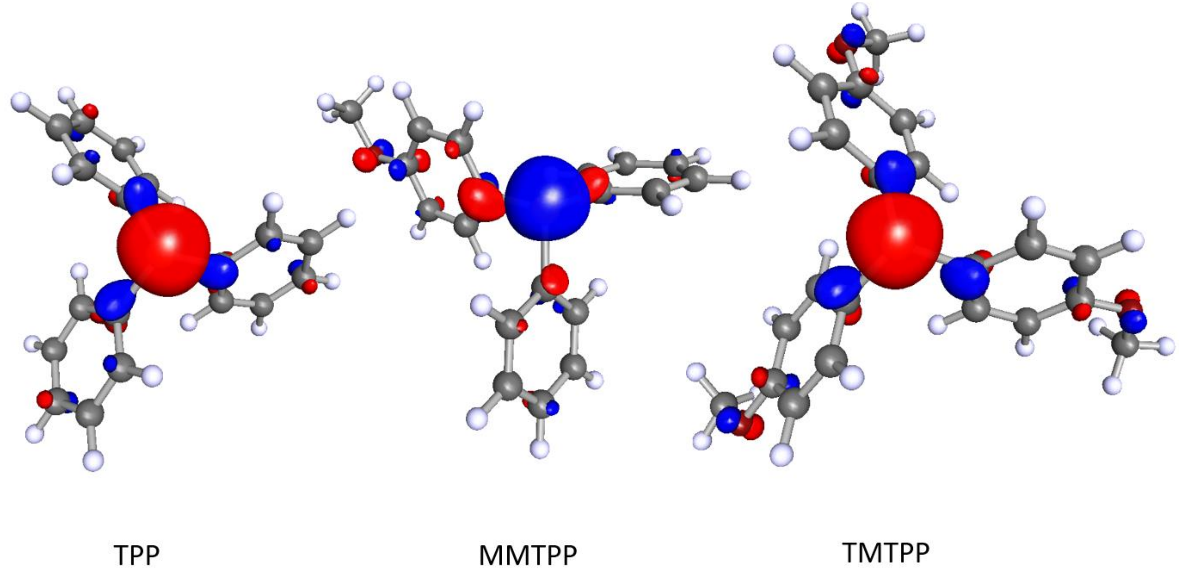

Figure S23: Visual representation of the HOMOs of TPP, MMTPP and TMTPP, calculated on B3LYP/def2-TZVPPD level

Table S6: Theoretical data for TPP, MMTPP, TMTPP, MCA values from Ref.5, pK_a values from Ref.6, HOMO and SOMO energies were calculated on B3LYP/def2-TZVPPD level

	MCA [kJ/mol]	pK_a	HOMO [eV]	SOMO [eV]
TPP	+618.7	1.31	-5.911	-9.596
MMTPP	-	2.85	-5.728	-9.175
TMTPP	+651.0	4.20	-5.418	-8.589

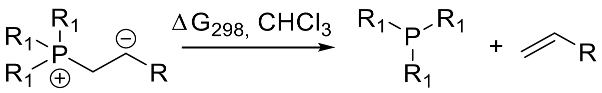

Scheme S1: Reaction for the Michael acceptor affinity (MAA) of a phosphine

Table S7: Calculated Michael acceptor affinities (MAA in kJ/mol) for phosphines PMe_3, TPP, MMTPP and TMTPP and Michael acceptors acrylonitrile, acrylamide and divinyl sulfone on B3LYP/def2-TZVPPD level

	R = acrylonitrile	R = acrylamide	R = divinyl sulfone
R^1 = Me	-74.6	-78.5	-64.6
R^1 = Ph	-96.4	-103.9	-85.6
R^1 = 2-(CH_3O)C_6H_4	-94.2	-103.9	-84.9
R^1 = 2,4,6-(CH_3O)$_3C_6H_2$	-87.7	-95.3	-76.7

References:

1 Bergbreiter, D.E.; Yang, Y.-C.; Variable-Temperature NMR Studies of Soluble Polymer-Supported Phosphine-Silver Complexes, *J. Org. Chem.*, **2010**, *75*, 873-878.
2 Heller, B.; Sundermann, B.; Buschmann, H.; Dexler, H.-J.; You, J.; Holzgrabe, U.; Heller, E.; Oehme, G.; Photocatalyzed [2+2+]-cycloaddition of nitriles with acetylene: an effective method for the synthesis of 2-pyridines under mild conditions, *J. Org. Chem.*, **2002**, *67(13)*, 4414-4422.

3 Luu, H.-T.; Wiesler, S.; Frey, G.; Streuff, J.; A Titanium(III)-Catalyzed Reductive Umpolung Reaction for the Synthesis of 1,1-Disubstituted Tetrahydroisoquinolines, *Org. Lett.*, **2015**, *17(10)*, 2478-2481.
4 Golobokova, T. V.; Pokatilov, F. A.; Proidakov, A. G.; Vereshchagin, L. I.; Kizhnyaev, V. N.; Synthesis of polynuclear azoles linked by ether tethers, *Russ. J. Org. Chem.*, **2013**, *49(1)*, 130-137.
5 Lindner, C.; Tandon, R.; Maryasin, B.; Larionov, E.; Zipse, H. Cation Affinity Numbers of Lewis Bases. *Beilstein J. Org. Chem*. **2012**, *8*, 1406-1442.
6 calculated using the pKa prediction platform (neural network result for solvent H2O) available at www.pka.luo-group.com see: Yang, Q.; Li, Y.; Yang, J.-D.; Liu, Y.; Zhang, L.; Luo, S.; Cheng, J.-P. Holistic Prediction of pKa in Diverse Solvents Based on Machine Learning Approach, *Angew. Chem. Int. Ed.* **2020**, *59*, 19282-19291.
